# Expression, Localization and Actions of Galectin-3: Implications in the Pathophysiology and Therapy of Cardiovascular Disease

**DOI:** 10.3390/ijms27135782

**Published:** 2026-06-26

**Authors:** Xiao-Jun Du, Gang She, Zheng-Da Pang, Yi Zhang, Xiu-Ling Deng

**Affiliations:** 1 Department of Physiology and Pathophysiology, College of Basic Medical Sciences, Xi’an Jiaotong University Health Science Center, Xi’an 710061, China; shegang@xjtu.edu.cn (G.S.); pangzd@mail.xjtu.edu.cn (Z.-D.P.); zhangyixjtu@xjtu.edu.cn (Y.Z.); dengxl@xjtu.edu.cn (X.-L.D.); 2 Cardiometabolic Innovation Center, Ministry of Education, Xi’an 710061, China; 3 Experimental Cardiology, Baker Heart and Diabetes Institute, Melbourne, VIC 3004, Australia

**Keywords:** galectin-3, Lgals3, transcriptional regulation, glycoconjugates, post-translational modification, metabolism, biomarker, cardiovascular disease

## Abstract

Research in the last two decades has well established galectin-3 (Gal3), a member of the lectin family, as a clinical biomarker and mediator of cardiovascular as well as other diseases. Gal3 contributes to progression of diseases by promoting pathological components, including inflammation, fibrosis, cell death or proliferation, and metabolic remodeling, and hence forms an ideal therapeutic target. Notably, nearly all Gal3 inhibitors that are currently under intensive pre-clinical and clinical testing target carbohydrate recognition/binding domain (CRD) of Gal3 molecules. Whereas the role of Gal3 in cardiovascular disease (CVD) has been well established, research on Gal3 in cancer or immunology has been leading the frontiers in this discipline. Therefore, it is important to have an integrated understanding on the biology and pathophysiology of Gal3 in a spectrum of pathological conditions. This review describes current findings from studies on diverse disease conditions and examines the role of Gal3 in the pathogenesis of diseases focusing on its transcription, post-translational modifications, intracellular dynamics, extracellular exporting, and interactions with a variety of signaling molecules. By bridging findings from different disciplines on the role of Gal3 in diseased settings, we explore the diverse anti-Gal3 strategies in addition to inhibition of CRD binding and highlight the significance of interventions targeting the transcription and post-translational modifications of Gal3, as well as intracellular actions of Gal3. At the end of this review, we provide perspectives for future research and therapeutic implications in CVD.

## 1. Role of Gal3 in Cardiovascular Disease and Knowledge Gap

**Figure 1:**
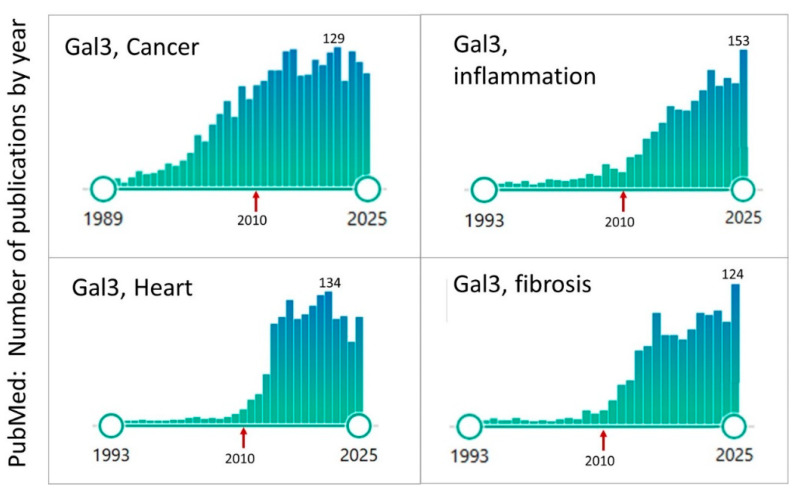
Number of publications per year in different research fields on galectin-3 (Gal3). The graphs show annual publications up to end of 2025 through PubMed search of Gal3 and diseased conditions of cancer, inflammation and fibrosis as well as the heart. Note that, judged by publication numbers, research on Gal3–cancer preceded other areas by approximately 20 years.

Galectin-3 (Gal3) belongs to the lectin family of β-galactoside-binding proteins. In the last two decades, accumulating studies have provided evidence for Gal3 as a disease mediator as well as a useful clinical biomarker for cardiovascular diseases (CVDs), including acute or chronic heart failure [1,2,3,4,5,6], arrhythmias [7] and systemic or pulmonary hypertension [8,9] and fundamental pathological processes like inflammation, fibrosis, atherosclerosis and thrombosis [10,11,12,13,14]. Therefore, Gal3 is now widely regarded as a therapeutic target [5,9,12,15]. Significant research effort has been devoted to the development of Gal3 inhibitors, mostly targeting carbohydrate recognition/binding domain (CRD) for blockade of Gal3 binding to its endogenous ligand glycoconjugates including glycoproteins and glycolipids [6,10,12,16,17] that account for approximately 50–70% of total proteins or lipids. In the last decade, the roles of Gal3 in CVD have been the subject of numerous review articles. However, few review articles on Gal3 have incorporated, in a significant proportion, findings from research on non-cardiovascular diseases, especially cancer. This could be a limitation given that research on the roles of Gal3 in cancer [18,19,20] or immunity [10,13] has pioneered research in this discipline and contributed significantly to our understanding of Gal3 molecular biology (Figure 1). In fact, investigators in the field of protein biochemistry or cancer pioneered research on Gal3 since the 1980s, being well ahead of research on the role of Gal3 in CVD, inflammation and fibrosis (Figure 1). Accordingly, in this review we also refer to publications of non-cardiovascular research in order to provide an updated and integrated summary of research findings on Gal3 and diseases.

### 1.1. Adverse Cardiovascular Actions of Gal3

Numerous studies have shown that Gal3 is able to bind to glycoconjugates localized in cytoplasm, cellular membrane or extracellular matrix (ECM). Gal3 influences multiple biological processes including cell–cell adhesion, cell–matrix interactions, activation of macrophages or fibroblasts, angiogenesis, cancer metastasis, apoptosis through its regulation of a diverse cellular signaling pathways, plasma membrane receptors or gene transcription [6,10,13,21,22,23]. Gal3 is currently regarded as a prognostic biomarker as well as a contributor to pathological conditions including heart disease, arteriosclerosis, neurological and inflammatory diseases and cancer [2,6,10,13,24,25,26,27,28]. Circulating Gal3 level acts as a prognostic biomarker but its value is significantly influenced by the capacity of renal clearance [4,29], given that Gal3 in blood circulation undergoes a rapid renal clearance [30]. Hence, circulating Gal3 might have a brief half-life and exert short-lasting biological actions mainly on circulating immune cells or vascular endothelial cells [13]. Numerous pre-clinical studies have shown that Gal3 is also a pivotal mediator promoting key pathological processes of inflammation, fibrosis and cell behavior [2,14]. There is strong evidence that inhibition of Gal3 by either Gal3 inhibitors or Gal3 gene knockout (*Lgals3*-KO) is beneficial [6,12,24]. In a mouse model of dilated cardiomyopathy (DCM), significant upregulation of cardiac Gal3 is associated with augmented pro-fibrotic and pro-apoptotic signaling [31], and deletion of the *Lgals3* gene in this model suppressed the expression levels of fibrotic and apoptotic genes [32]. Under conditions of ischemia–reperfusion, *Lgals3*-KO is protective against myocardial injury and mitochondria-mediated cardiomyocyte apoptosis [33]. Upon diabetic metabolic challenge, Gal3 is found to mediate cardiomyocyte death, fibrosis and cardiac remodeling by interfering with the activity of Akt [34]. Elevated circulating levels of Gal3 also mediate augmented platelet aggregation and thrombosis [35].

Numerous studies also provided strong evidence for a pivotal role of Gal3 in vascular diseases. Dysfunction of vascular endothelial cells plays a central role in the development of systemic hypertension or pulmonary arterial hypertension. In the mouse model of hypertension induced by angiotensin II infusion, we found that *Lgals3* deletion abolished the upregulated levels of CD68, intracellular adhesion molecule (ICAM), vascular cell adhesion molecule (VCAM), interleukins (ILs) and monocyte chemoattractant protein-1 (MCP-1), together with normalization of blood pressure and endothelium-mediated vasodilation [36]. In patients with pulmonary artery hypertension, elevated blood levels of Gal3 predict poor prognosis [8,37,38,39], and Gal3 is shown to mediate pulmonary vascular remodeling that involves endothelial-to-mesenchymal transition (EMT), proliferation of vascular smooth muscle cells (VSMCs) and vascular fibrosis [8,37,38,39]. In atherosclerotic arteries, Gal3 facilitates formation of foam cells through VSMCs, thereby increasing regional plaque lipid accumulation [40]. In high-cholesterol-feeding animals, increased lipid loading in blood vessels can be effectively prevented by treatment with Gal3 inhibitor GB1107. Increased aortic levels of Gal3, largely derived from M1 macrophages, promotes expression of a panel of inflammatory cytokines (IL-1β, IL-8, tumor necrosis factor-α, TNF-α), matrix metalloproteinase-9 (MMP-9) and Fas, by which Gal3 induces VSMC apoptosis and destruction of extracellular matrix (ECM), and eventually formation of aortic aneurysm [41]. Therapeutically, Gal3 inhibitors, mostly targeting CRD, have been developed and tested in a number of animal models of CVD (Table 1), albeit pre-clinical and clinical studies yielded inconsistent findings [12,16,17,32].

### 1.2. Knowledge Gap and Aims of This Review

Despite the significant progress over the last two decades in research on Gal3 and CVD, significant knowledge gaps exist regarding biological actions of Gal3 and therapeutic approaches to inhibit Gal3 activity. First, our understanding about the actions of Gal3 is largely limited to extracellular- or membrane-localized Gal3 for cell–cell, cell–matrix or cell–pathogen interactions, whilst the intracellular localization and actions of Gal3 are only partially understood. Relevant to this is the intracellular transportation and extracellular exporting in cardiovascular tissues/cells, processes that have been much less studied. Second, whereas CRD binding of Gal3 to glycoconjugates has been well known, the CRD-independent actions of Gal3 have been superficially investigated. Similarly, the transcriptional regulation of Gal3 is only partially defined. Theoretically, all these aspects hold the promise to form therapeutic targets against Gal3. In this review, we will discuss current research findings on the transcription, translation, post-translational modifications (PTMs) and intracellular biological actions of Gal3 including mitochondrial metabolism. Third, numerous studies on CVD in clinical and experimental settings have shown significant increases in the tissue and/or circulating levels of Gal3 and the correlation between Gal3 levels and disease markers or the severity including pathological cardiovascular remodeling and risk of adverse cardiac events [6,12,17,28,42], albeit the signaling mechanism is only partially defined. These findings suggest the potential of targeting signaling pathway(s) that mediate Gal3 upregulation for the treatment of CVD. To achieve this goal, good understanding of transcriptional regulation of Gal3 expression is essential. In this review, the mechanisms(s) that govern the transcriptional expression of Gal3, PTM, and the nuclear localization and actions are particularly discussed. Based on these research findings, we propose at the end the therapeutic approaches independent of CRD-binding inhibition to halt the adverse actions of Gal3 in CVD. Fourth, research has been relatively limited on Gal3 molecular biology in cardiovascular tissues/cells. As displayed in Figure 1, studies conducted in cancer pioneered Gal3 research relative to other disciplines and have contributed significantly to our understanding of Gal3. Research in a range of cancer cells has well established Gal3 as an important oncogenic regulator of cancer cell behavior impacting cancer cell growth, transformation, apoptosis, angiogenesis, invasion and metastasis [43,44,45,46,47]. Such a divergent influence of Gal3 on cancer cells derives from its intra- or extracellular localization where it interacts with different binding partners [18,19,44]. Thus, in the current review, we include studies on different diseased models to provide a pan-disciplinary coverage of Gal3 biology and pathobiology with the view of combating the adverse actions of Gal3 in CVD. Finally, our understanding is limited with regard to the efficacy of CRD-targeted Gal3 inhibitors as well as CRD-independent actions of Gal3. The pleotropic functions of Gal3 are well known to be dependent, in a large part, on its CRD–glycoconjugate-binding capacity. Pre-clinical research has employed interventions such as *Lgals3*-KO, chemicals that compete with glycoconjugates for CRD-binding sites of Gal3, i.e., modified citrus pectins (MCPs) and derivatives, or small molecular chemicals with higher selectivity and affinity for Gal3 (Table 1) [17]. There are reports showing that CRD-based Gal3 inhibitors, when tested in a virtual intracellular environment, differ significantly in their capability of accumulation within intracellular organelles, which is independent of their CRD-binding affinity [20,48]. Whereas numerous studies have reported similar effectiveness of Gal3 inhibitors versus *Lgals3*-KO in diverse diseased settings [6,12,15,17,49,50,51], there is a study showing in a mouse DCM model that *Lgals3*-KO, by crossbreeding DCM with *Lgals3*-KO strains, significantly ameliorated cardiac fibrosis, ventricular remodeling and dysfunction, whereas chronic treatment with MCPs failed in achieving health benefits [32]. Thus, the inconsistency in the efficacy of CRD-based inhibitors versus *Lgals3*-KO might be explained by the fact that the intracellular actions of Gal3 are simultaneously suppressed by *Lgals3* gene deletion.

## 2. Transcriptional Regulation of Expression of Lgals3

### 2.1. Structure of Galectin-3 Gene and Transcriptional Regulation

**Figure 2:**
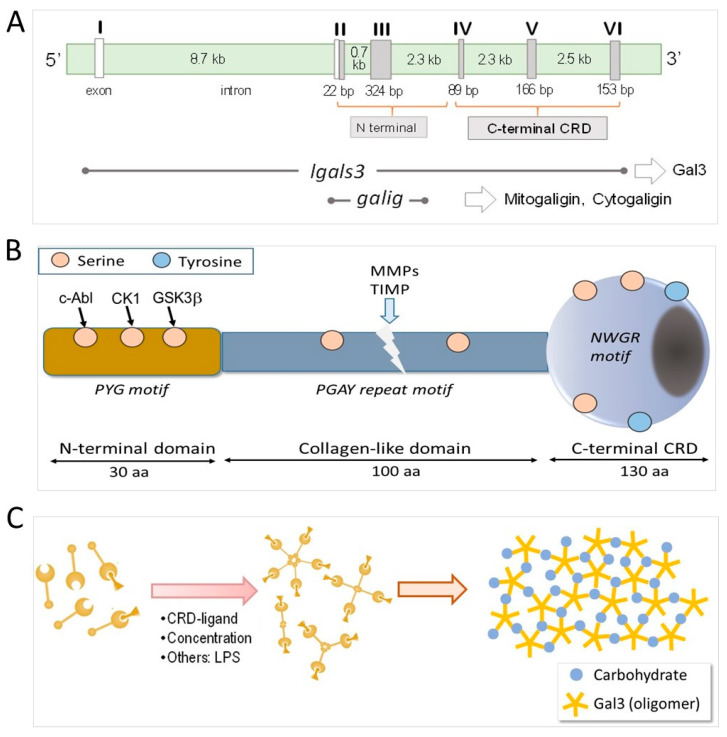
Structure of *Lgals3* gene or Gal3 protein and its oligomerization and formation of Gal3–glycoconjugate lattices. (**A**) Located in chromosome 14 locus q21-22, *Lgals3* gene is composed of six exons and five introns. The sequence encoding N-terminal domain is localized in exon III, and the sequence encoding CRD is in exon V (human) or exons IV, V and VI (mouse). Depicted also is the internal gene *Galig* that encodes mitogaligin and cytogaligin, proteins independent of Gal3. (**B**) Gal3 protein structure containing three domains. Also indicated are serine/tyrosine sites for phosphorylation, the site for cleavage by matrix metalloproteinases (MMPs) and several motifs critical in protein–protein interactions. (**C**) Gal3 oligomerization into dimers, trimers, tetramers or pentamers and formation of Gal3–glycoconjugate lattice on cellular membrane. The plot represents the view of lattices from the surface of plasma membrane. This structure has been implicated in regulating numerous biological functions such as cell signaling, cell migration and cell adherence. Formation of Gal3/glycoconjugate lattices on the plasma membrane could influence the stability, localization, trafficking, clustering and activity of cell surface receptors and other proteins.

The Gal3 gene, *lgals3*, is situated on chromosome 14 locus q21-22 and composed of six exons and five introns spanning about 12 kb in the mouse and 17kb in humans (31 kDa) (Figure 2A). In human and murine *Lgals3*, the sequence encoding the N-terminal domain is similarly localized in exon III, whilst the sequence encoding CRD is located in exon V in humans but in exons IV, V and VI in the mouse (Figure 2A). *Lgals3* is ubiquitously expressed in multiple human tissues and cells. *Lgals3* is constitutively expressed in fibroblasts, endothelial cells, neutrophils, monocytes and macrophages and, in certain cell types like lymphocytes or cardiomyocytes that normally do not express Gal3, the expression can be induced upon pathological stimuli.

Studies have revealed the presence of an internal gene, *Galig*, that is embedded within the *Lgals3* gene and encodes distinct proteins. Guittaut et al. identified two transcripts arising from the *Galig* gene in human [52] and found that two overlapping alternative open reading frames (ORF1 and ORF2) of the *Galig* gene are translated, leading to two proteins independent of Gal3 (Figure 2A). One protein is termed mitogaligin that mainly localizes to mitochondria and also the nucleus. Another protein encoded by *Galig* is cytogaligin that localizes to the cytosol. Mitogaligin is a 96-amino-acid peptide and its mitochondrial localization is due to its internal sequence of residue 31–47 [53,54]. Studies in cultured cells found that mitogaligin destabilizes mitochondrial membranes via interaction with cardiolipin, a class of mitochondria-rich phospholipid [55], leading to the release of mitochondrial cytochrome *c*, mitochondrial aggregation, nuclear condensation and apoptotic cell death [53,54,56]. Indeed, overexpression of GALIG proteins induces cell apoptosis. Little is known about the cardiovascular actions of both proteins. α-Synuclein is a neuronal protein highly expressed in the brain and accumulation of α-synuclein can form protein deposits as Lewy bodies in neurons in patients with dementia or Parkinson’s disease. Haddad et al. found that overexpression of cytogaligin lowers the level of α-synuclein aggregation, suggesting an interaction of cytogaligin with α-synuclein in neurons [56].

### 2.2. Transcription Factors and Epigenetic Factors Regulating Lgals3 Transcription

**Figure 3:**
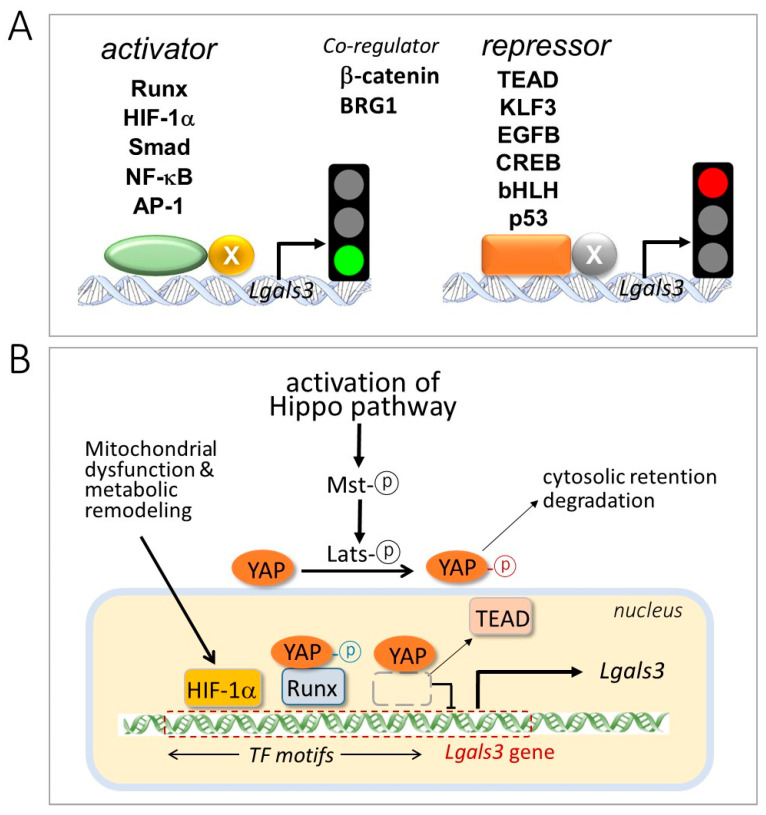
Transcriptional regulation of the expression of *Lgals3*. (**A**) Listed are known transcription factors that either activate or suppress the expression of *Lgals3*. X represents epigenetic factors. (**B**) Activation of Hippo pathway in cardiomyocytes leads to enhanced expression of *Lgals3* through different mechanisms including inhibitory phosphorylation of YAP (Ser-127) that impedes its nuclear entry. Data is based on the findings from transgenic mice with cardiomyocyte-restricted expression of Mst1 leading to activated Hippo pathway signaling. Abbreviations: YAP: Yes-associated protein; Mst1: mammalian sterile 20-like serine/threonine kinase 1; Runx: Runt-related transcription factor; HIF-1α: hypoxia-inducible factor-1α; AP-1: activator protein-1; NF-κB: nuclear factor kappa B; BRG1: Brahma related gene1, a SWI/SNF family member; TEAD, TEA-domain family member; KLF3: Krüppel-like factor3; EGF: epidermal growth factor; bHLH: basic helix–loop–helix transcription factor; CREB: cAMP-responsive element-binding protein, TF: transcription factor.

The promoter region of the human *LGALS3* gene contains several regulatory binding motifs: five sites for putative SP-1 binding (GC boxes), five sites for cAMP-responsive element-binding protein (CREB), four for AP-1-like and one for AP-4-like sites, two for nuclear factor-κB (NF-κB)-like sites, one for the sis-inducible element (SIE) and one basic helix–loop–helix (bHLH) consensus sequence [57]. The sequence CTCTTTC (or its variant GTCTTTC) in the human *LGALS3* promoter represents the transcription-factor-binding motif leading to significant activation of *Lgals3* expression [58]. Studies have identified a number of transcription factors that are able to bind to the *Lgals3* promoter, thereby regulating transcription in a variety of cell types (Figure 3). In cancer cells, enhanced *Lgals3* expression is tightly related to the activity of AP-1, Wnt/catenin signaling [59], RUNX1/2 [60,61], NF-κB [62,63,64], hypoxia-inducible factor-1α (HIF-1α) [65], myocardin-related transcription factor (MRTF) [40], Smad [66] and β1-integrins [67]. Runx2 is able to bind to the *Lgals3* promoter either upstream (motif TCTGGT) or downstream (CACCGCC) [61]. There are reports showing that HIF-1α mediates upregulation of Gal3 in non-cardiovascular tissues [68,69,70]. There might be complex crosstalk among transcription factors, acting either as activator or repressor, in the regulation of *Lgals3* expression such as Krüppel-like factor 3 (KLF-3) [71], p53 [47,72] and CREB of AMP-activated protein kinase (AMPK) signaling (Figure 3A). *Lgals3* expression was enhanced by MMP-12 or by other pro-inflammatory cytokines and chemokines [73] albeit the regulatory mechanism remains undefined.

Epigenetic regulation of *Lgals3* expression has also been described. Brahma related gene 1 (BRG1) is a subunit of the mammalian SWI/SNF complex that regulates chromatin accessibility and gene transcription by remodeling histone DNA via interacting with other epigenetic machinery or non-coding RNAs. Li et al. showed, in cultured hepatocytes, that treatment with fatty acid palmitate or lipopolysaccharides (LPSs) stimulated expression of *Lgals3*, which was abolished by depletion of BRG1 [74]. They also observed in several models of hepatic injury that BRG1 deletion in vivo effectively suppressed Gal3 expression [74]. Further analysis revealed that BRG1 interacted with AP-1 to bind to the proximal *Lgals3* promoter and activate transcription. Mechanistically, the methylation/demethylation status of *Lgals3* DNA surrounding the *Lgals3* promoter appeared to be a rate-limiting step in BRG1-mediated activation of *Lgals3* transcription. BRG1 recruits the DNA 5-methylcytosine dioxygenase TET1 to the *Lgals3* gene, thereby promoting active DNA demethylation and *Lgals3* transcription, and TET1 silencing in cultured hepatocytes abrogated induction of *Lgals3* expression stimulated by LPS and palmitate. Whereas DNA methylation of the *Lgals3* promoter silences *Lgals3* transcription in epithelial cells, expression of β1-integrin causes its demethylation, revivifying its transcriptional activity [67]. Similarly, in pituitary cancer cells *Lgals3* transcription is repressed by DNA methylation of the promoter region [75]. In a model of myocardial fibrosis induced by radiation injury, upregulation of *Lgals3* is induced by H3K27 acetylation in the promoter region, which is associated with reduced expression of Sirtuin2 (protein deacetylase, Sirt2) and upregulation of fibrotic genes [76]. Conversely, overexpression of Sirt2 suppressed expression of *Lgals3* and fibrotic genes and reduced organ fibrosis, effects that were reversed by administration of Gal3 [76]. In a recent study Song et al. revealed a role of deacetylase Sirt6 in regulating *Lgals3* expression in mast cells prepared from a murine model of diet-induced obesity, where Sirt6 acts as a *Lgals3* suppressor through mediating histone deacetylation at the promoter region, and the loss of this inhibitory regulator results in enhanced *Lgals3* expression and activation of M1-type macrophages with exacerbated obesity and inflammofibrosis [77]. In a model of allergic airway inflammation, use of inhibitor of histone deacetylase8 (HDAC8) suppressed expression of Gal3 together with inhibition of macrophage infiltration [78].

### 2.3. Regulation of Lgals3 Transcription by TGF-β/Smad or NF-κB Signaling Pathway

**Figure 4:**
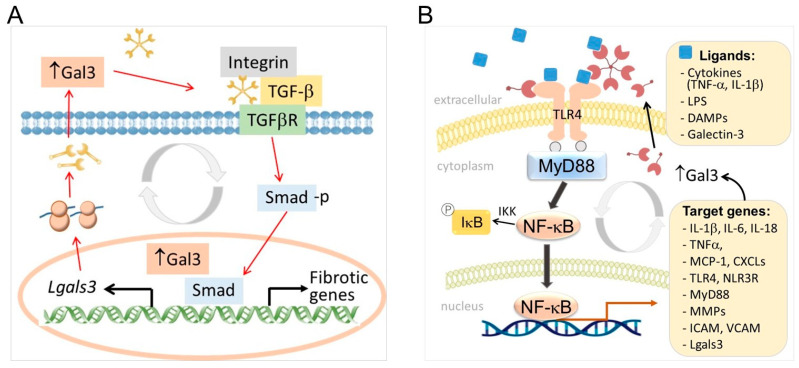
Amplification of TGF-β or NF-κB signaling pathway by Gal3 leading to enhanced inflammation and fibrosis. (**A**) Extracellular Gal3 acts as a scaffold protein facilitating the complex formation of integrin/TGF-β/TGFβR, that facilitates TGF-β downstream signaling. The TGF-β/Smad pathway mediates upregulation of *Lgals3* and Gal3, forming the TGF-β/Gal3 positive feedback loop (curved arrows). (**B**) The Gal3/TLR/MyD88/NF-κB pathway forms a feedback signaling loop between Gal3 expression and NF-κB pathway leading to upregulation of various inflammatory cytokines, chemokines and adhesion and stimulatory molecules including *Lgals3*. Gal3 acts as a ligand for TLR or a facilitator for the interaction of TLR and its canonical ligands (cytokines) leading to activation of the TLR/MyD88 cascade. Abbreviations: IKK: inhibitor of kappa B kinase; NF-κB: nuclear factor kappa-light-chain-enhancer of activated B cells; TLR: Toll-like receptor, LPS: lipopolysaccharide; DAMP: danger-associated molecular pattern; NLR; Node-like receptor; MyD88: myeloid differentiation primary response 88.

Research has well established the role of Gal3 in promoting inflammation and fibrosis, in which the transforming growth factor-β (TGF-β) or NF-κB signaling pathways play major roles (see Section 6.1 and Section 6.2). There exist reciprocal interactions between Gal3 and both TGF-β and NF-κB signaling pathways. Through simultaneous CRD binding of Gal3 to integrins and TGFβRII localized on the surface of cells, Gal3 is able to facilitate the activation of the TGF-β1/TGF-receptor (TGFβR)/Smad signaling that is known to be critical for fibroblast activation [66]. Importantly, *Lgals3* expression per se is upregulated by Smad, forming a pro-fibrotic feedback loop (Figure 4A). Besides promoting the fibrotic profile, this signaling loop stabilizes atherosclerotic plaques by attenuating the inflammatory phenotypes of macrophages [73]. Such action of Gal3 is enhanced under diseased conditions with increased TGF-β1 level and can be attenuated by *Lgals3* gene deletion [32]. A similar positive feedback signaling loop also exists between upregulated *Lgals3* and pro-inflammatory NF-κB signaling that augments expression of *Lgals3*/Gal3 as well as membrane receptors or cofactors of the NF-κB signaling pathway (Figure 4B). Studies have shown that this mechanism contributes to the activation of the Gal3/Toll-like receptor (TLR)/MyD88/NF-κB signaling axis and facilitation of inflammatory responses in a range of diseased settings or immune cells [64,73,79,80,81,82,83,84,85,86]. There is also report of regulation of Gal3 expression by minerocorticoid receptors. Lin et al. observed in patients with primary aldosteronism that, in addition to hypertension, the blood level of Gal3 is markedly elevated, which was corrected by adrenalectomy [87]. Further experiments in vitro found that treatment of macrophage cell lines with aldosterone enhanced expression of *Lgals3*/Gal3, action dependent on the minerocorticoid receptor, PI3K/Akt and NF-κB signaling [87].

### 2.4. Regulation by the Hippo-YAP Signaling Pathway

The Hippo signaling pathway plays a key role in the regulation of cell differentiation and organ development [88]. The core components of the Hippo pathway include two upstream kinases, mammalian sterile 20-like kinase1 (Mst1) and large tumor suppressor homolog (Lats), and downstream transcriptional co-regulator yes-associated protein (YAP). Nuclear YAP regulates expression of numerous genes by interacting with various transcription factors like the TEA-domain family member (TEAD). Phosphorylation of YAP at different sites regulates its intracellular localization. Mst1/Lats induce YAP Ser-127 site phosphorylation (Ser-127-pYAP) that inhibits YAP nuclear translocation together with cytoplasmic retention and subsequent degradation. The significance of the Hippo pathway in the development of cardiomyopathy and heart failure is indicated by two lines of research findings. Experimentally, cardiomyocyte-restricted genetic modifications in mice with either Hippo pathway activation (e.g., Mst1-cTG) or YAP/TEAD1 inactivation (e.g., YPA-cKO, TEAD1-cKO) result in a rapid onset and progression of a DCM phenotype [89,90,91,92]. Clinically, several studies using human cardiac tissues from patients with DCM, ischemic or arrhythmic cardiomyopathy or heart failure provided evidence for Hippo pathway activation measured by upregulation and activation of Mst1 and Lats, YAP inactivation (increased Ser-127-pYAP), or TEAD1 downregulation [93,94,95,96]. Thus, cardiac Hippo pathway activation is closely associated with the onset and development of cardiomyopathy and heart failure.

Several pieces of evidence indicate that Hippo pathway signaling regulates the expression of Gal3 in cardiomyocytes (Figure 3B). First, activation in cardiomyocytes of the Hippo pathway leads to remarkable upregulation of *Lgals3* and Gal3 at mRNA and protein levels, as shown in the Mst1-cTG strain of mice [31,32]. The signaling mechanism remains unclear but very likely multifactorial. In Mst1-cTG mouse hearts, there is a 2.5-fold increase in protein abundance of HIF-1α [91]. In fact, HIF-1α is known to upregulate Gal3 expression in cardiac or non-cardiac tissues or cells (Figure 3) [65,68,69,70,97]. In the Mst1-cTG mouse heart, certain galectin members (Gal1, 8 and 9) were also upregulated by 1.3- to 2-fold, the scale being far less relative to that of *Lgals3*/Gal3 (by 60–80-fold) [32]. Importantly, upregulation of Gal3 occurs in the very early phase of DCM in this Mst1-cTG model, highlighting its causal role in the onset of the cardiomyopathy phenotype [91]. Second, drugs like verteporfin that inhibits nuclear YAP-TEAD1 interaction similarly increased Gal3 expression in the heart [96]. Third, in cultured cell models, YAP gene knockdown using siRNA resulted in upregulation of *Lgals3* and Gal3. Collectively, these findings suggest a working mode in which cardiomyocyte YAP exerts inhibitory regulation of Gal3 expression, and inactivation of YAP, either due to Hippo pathway activation (e.g., Mst1-cTG) or by verteporfin or YAP knockdown, invalidates this inhibitory action leading to Gal3 upregulation (Figure 3B). In addition to TEAD family members, YAP also interacts with many transcription factors including p73, ErbB4, T-box transcription factor 5 (TBX5), Smads, HIF-1α, Runx (at different levels) and AP-1 [98]. Research on different tissues or cancer cells has shown that Runx-2 facilitates *Lgals3* expression [60,74,99]. Phosphorylation by Lats not only leads to suppressed YAP-TEAD interaction but is also associated with an enhanced complex formation of Ser-127-pYAP-Runx, which potentially facilitates Gal3 expression (Figure 3B).

However, unlike the regulation by Hippo-YAP signaling of Gal3 expression in the myocardium, in the setting of angiotensin-II-induced hypertension, activation of YAP in vascular endothelial cells is able to enhance Gal3 expression [36]. This process is initiated by AT1R/Gq stimulation and subsequent activation of tyrosine kinase Src, which mediates YAP phosphorylation at Tyr-357 together with dephosphorylation at Ser-127, leading to enhanced YAP nuclear localization. In fibroblasts, YAP is known to promote Gal3 expression and cell activation contributing to organ fibrosis [100,101].

### 2.5. Regulation by β-Adrenergic Signaling

Zhao et al. first reported that activation of cardiac β-adrenergic receptors (βARs), either by pharmacological or genetic means, is potent in upregulating the expression of *Lgals3*/Gal3 at mRNA and protein levels [31]. They showed that administration of βAR agonist isoproterenol to mice led to a rapid and dose-dependent upregulation of Gal3 in the heart, an effect that can be abolished with selective or non-selective β-blockers (atenolol, ICI-118551, carvedilol and propranolol). In keeping with these findings by the use of β-agonists, transgenic mice with cardiomyocyte-specific overexpression of β_1_AR or β_2_AR exhibited 10–20-fold upregulation of the Hippo kinase Mst1 as well as Gal3 [31,102,103,104]. βAR signaling in mediating such coupling is dependent on Gs-protein, adenylyl cyclase and cAMP-protein kinase-A. Further studies have revealed the coupling of βAR and Hippo pathway signaling [12,31]. This finding in the heart in vivo is in keeping with a previous report demonstrating that the Hippo pathway is under regulation by G-protein coupled receptor (GPCR) signaling [105]. Interestingly, there is in vivo evidence for Hippo pathway dependency of isoproterenol-induced Gal3 upregulation in the heart. Whilst the Mst1-cTG mice responded to isopretorenol with a 10-fold higher increase in Gal3 expression relative to that of wild-type control mice, this response was largely prevented in mice with cardiomyocyte-restricted expression of a dominant-negative mutant Mst1 gene (dnMst1-cTG) [31]. Other signaling mechanisms are also potentially involved in the βAR-Hippo pathway signal coupling leading to Gal3 upregulation, including activation of mitogen-activated protein kinase (MAPK), Src, Ca^2+^-RhoA, as well as remodeling of the cytoskeleton where YAP acts as a mechanical sensor [106].

### 2.6. Regulation by Other Factors or Autoregulation

There have been an increasing number of reports on the regulation of *Lgals3* transcription by metabolites or inflammatory molecules in different tissues or cell types. There are studies showing that fatty acid (palmitate) induced Gal3 expression in hepatocytes [74] and phorbol myristate acetate (PMA) stimulates Gal3 expression in monocytes/macrophages [27]. In adipocytes, expression of Gal3 is regulated by fatty acids as well as cytokines [107]. In reparative M2 macrophages isolated from the infarcted mouse heart, IL-10 promoted *Lgals3*/Gal3 expression and tissue repair, which was dependent on STAT3 tyrosine phosphorylation [108]. In white and brown adipocytes, TNF-α reduces but IL-6 and fatty acids (almitate, oleate and linoleate) elevate Gal3 levels in supernatants and cells in a concentration-dependent manner [107]. Thus, upregulation of *Lgals3*/Gal3 in adipocytes by free fatty acids and IL-6 may contribute to higher Gal3 levels in patients with obesity. In blood vessels, exposure to high cholesterol increased de novo expression of Gal3, which is mediated by myocardin-related transcription factor A (MRTF) [40]. In cancer cells, *Lgals3* expression was upregulated by enhanced protein glycosylation (O-GlcNAcylation) albeit the precise mechanisms remain unclear [109]. *Lgals3*/Gal3 expression is also found to be regulated by Src, a non-receptor tyrosine kinase. In the mouse model of angiotensin-II-induced hypertrophy and fibrosis, upregulated expression of Gal3 was abolished by conditional Src-KO in vivo and in vitro [110]. In cultured monocytes, exposure to IL-4 alone strongly induced *Lgals3* at mRNA levels, while concomitant treatment with IL-4 and granulocyte–macrophage-colony-stimulating factor (GM-CSF) induced release of Gal3 [111].

There is evidence for nuclear Gal3 in mediating autoregulation of *Lgals3* transcription. Fei et al. reported that Gal3 released by bone marrow stromal cells enters lymphoblastic leukemia cells and is then transported into the nucleus, where Gal3 stimulates de novo transcription of *Lgals3* mRNA [112]. Such autoinduction of *Lgals3*/Gal3 expression contributes to chemotherapy resistance of leukemia cells. In models of multiple sclerosis, co-expression modular analysis revealed that Gal3 is co-expressed together with genes involved in the regulation of microglial cells, the main immune cells in the central nervous system, contributing to neuronal inflammation and damage [113]. Whether similar autoregulation exists in cardiovascular tissues/cells remains to be examined.

## 3. Post-Transcriptional Regulation of Lgals3 and Post-Translational Modifications of Gal3

Like any mRNA or pre-protein, the transcription of *Lgals3* and translation of Gal3 are subjected to complex regulation at different levels. *Lgals3* mRNA is known to be regulated by non-coding RNAs (ncRNA), mRNA modification and transportation from the nucleus into cytoplasm. The process of transcriptional regulation, maturation and localization of Gal3 is complex and also influenced by Gal3 chemical modulations, especially phosphorylation.

### 3.1. Regulation by Non-Coding RNAs

Numerous studies in CVD, cancer or other tissue/cells have provided direct and indirect evidence for the regulation by ncRNA of transcription or stability of *Lgals3* and Gal3 translation. In settings of CVD and cancer, a number of mRNAs are found to regulate levels of *Lgals3* or Gal3, either by upregulation (e.g., miR-9, miR-21, SNHG20, miR-199a-3p, miR-204-5p, miR-210-3p, miR-214, miR-335) [114,115,116,117,118] or downregulation (e.g., miR-1, miR-27, miR-27-3p, miR-185, miR-204-5p, miR-128, miR-128-3p, miR-204-5p, miR-299-5p, miR-322, miR-424-3p, miR-let-7d) [119,120,121,122,123,124,125]. In cardiac tissues from patients or animals subjected to ischemia–reperfusion, Rong et al. demonstrated that the expression of *Lgals3* was upregulated when miR-204-5p level was low and that the expression levels of miR-204-5p and *Lgals3* were negatively correlated [117]. Bioinformatics predicting interactions between mRNA and miRNA identified a few miRs (like miR-204-5p) and ncRNA (e.g., KCNQ10T1) regulating *Lgals3* expression. Notably, inhibition by miR204-5p was counter-regulated by KCNQ10T1, indicated by the finding that lowering KCNQ10T1 restores miR-204-5p level and simultaneously reduces *Lgals3* mRNA level. A recent study showed that lcRNA XRA8008038 upregulates expression of Gal3 under conditions of ischemia–reperfusion, thereby exacerbating cardiac injury [126]. In patients with acute heart failure complicated by diabetes, a clinical study revealed a significant and positive correlation between elevated circulating levels of miR-214 and *Lgals3* mRNA [115]. In patients with impaired heart function and poor prognosis, Vegter et al. observed a negative correlation between miR-199a-3p expression and Gal3 serum concentration at the 48th hour after hospitalization (r = −0.73; *p* < 0.001) [127], implying that expression and/or secretion of Gal3 are counter-regulated by miR-199-3p. Zhang et al. reported that *Lgals3* is a target of miR-27b through which miR-27b mediates cardioprotection against myocardial hypertrophy [33]. Other studies reported that levels of ncRNAs correlated positively (SNHG20, miR-21, miR-214) or negatively (miR-335, miR-1) with the level of *Lgals3* in a variety of heart diseases [128]. The regulation by ncRNAs of *Lgals3* expression holds the promise to be developed further as therapeutic targets to suppress elevated expression of Gal3 under diseased conditions.

### 3.2. Lgals3 mRNA Nuclear Exporting, Stability and Modification

**Figure 5:**
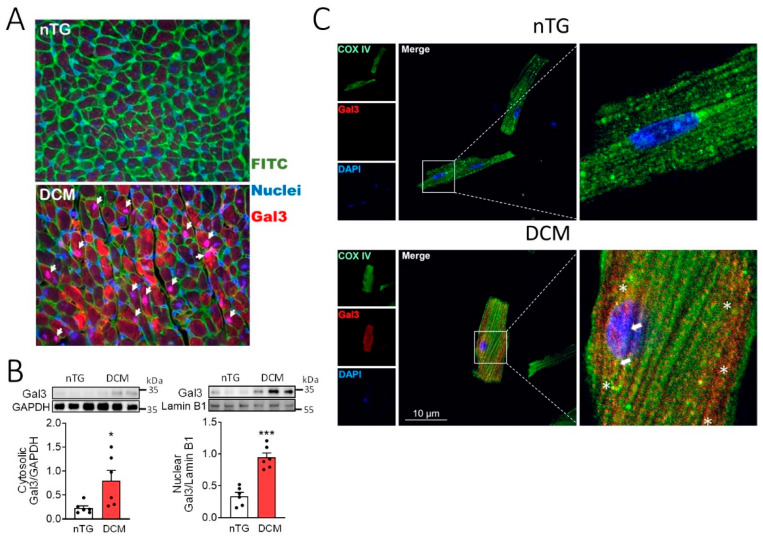
Nuclear localization of Gal3 in cardiomyocytes of transgenic Mst1-cTG mice with dilated cardiomyopathy (DCM). (**A**) Immunohistochemical images of the left ventricular myocardium of non-transgenic control (nTG) and transgenic DCM mice. Extracellular matrix (by wheat germ agflutinin, WGA), nuclei (by DAPI) and Gal3 were stained. Arrows indicate nucleus-localized Gal3 in cardiomyocytes. (**B**) Representative immunoblotting image of cytosolic or nuclear protein fraction from DCM mouse hearts. Dots represent individual cardiac samples. * *p* < 0.05, *** *p* < 0.001 vs. nTG. (**C**) Immunofluorescence confocal images of isolated adult cardiomyocytes of nTG and DCM mice. Arrows indicate nuclear Gal3 and asterisks indicate mitochondrial Gal3. Images are from authors’ previous publications with permission [32,131].

Transportation of *Lgals3* mRNA from the nucleus to cytoplasm requires a specific export receptor and protein complex forming an export machinery and interaction with the nuclear pore complex (NPC). The export process is also coupled to pre-mRNA modification (e.g., splicing) and maturation prior to cytoplasmic translation [129]. Emerging studies revealed that modification and stability of *Lgals3* mRNA influence its transcription. Zhang et al. found in mice and H9c2 cells that, upon pro-hypertrophic stimuli by either pressure overload or angiotensin II infusion, expression level of Gal3 increased by 3–4-fold, meanwhile in angiotensin-II-treated cells there was 70% reduction in the content of *Lgals3* with N6-methylaadenosine (m6A)*,* a RNA modification that reduces the stability of *Lgals3* mRNA [130]. Intervention using tanshinone IIA, a drug known to be vasoprotective against reactive oxygen species (ROS), inflammation or atherosclerosis, suppressed hypertrophy and Gal3 expression, together with increased content of m6A-*Lgals3*. These findings suggest that modification of m6A content of *Lgals3* mRNA holds the potential of targeting Gal3 expression. Whilst Gal3 protein level is very low in normal heart tissues, we found in Mst1-cTG mouse hearts a 60–80-fold increase in Gal3 protein level by ELISA, mainly in cardiomyocytes (Figure 5) [32,91]. At the mRNA level, there is approximately a 70-fold increase in *Lgals3* mRNA by RNA sequencing or RT-PCR. Our recent single-cell nuclear RNA-seq (snRNA-seq) showed a 15-fold increase in *Lgals3* mRNA level in cardiomyocytes (our unpublished observation). This difference in the expression level of *Lgals3* mRNA in whole tissue RNA-seq versus snRNA-seq of cardiomyocytes is likely explained by an active *Lgals3* nucleus-to-cytoplasm transporting machinery in cardiomyocytes of Mst1-cTG heart, which is critical for efficient Gal3 protein translation. This finding, if confirmed by further investigation, would implicate a therapeutic intervention to suppress *Lgals3* mRNA exporting and hence Gal3 protein biosynthesis.

### 3.3. Gal3 Structure, Oligomerization and Formation of Gal3 Lattices

Encoded by *Lgals3*, Gal3 contains about 130 amino acids and has a full molecular size of 25–35 kDa. Structurally, Gal3 is composed of three distinct domains: N-terminal domain, collagen-like domain containing proline–glycine-rich sequences, and a globular C-terminal domain containing the CRD site (Figure 2B). Gal3 protein is highly conserved among mammals with a homology over 95%. Gal3 is able to interact with numerous ligands either by CRD binding to glycoconjugates or through direct protein–protein interactions. A study on single-point mutations of amino acids within CRD revealed that the affinity and specificity of CRD–ligand binding depend on several amino acids, including R186, K176 and G182 [132]. Of them, R186S mutation abolishes ligand binding. While sharing a similar structure in the C-terminal CRD, Gal3 is unique relative to other galectin family members in its structure containing a collagen-like domain and N-terminal domain (Figure 2B). Gal3 oligomerization occurs in solution or on the plasma membrane. Using techniques of site-directed fluorescence labeling of Gal3 and fluorescence resonance energy transfer detection, Nieminen et al. found that Gal3 at physiological concentrations undergoes oligomerization on the surface of neutrophils following CRD binding to glycoconjugates [133]. Brewer et al. showed that, upon ligand binding in solution, high concentrations of Gal3 precipitate as oligomers, which is attributable to the self-association property of its N-terminal domain [134]. Specifically, Gal3 monomers, after binding to a ligand, can recruit additional Gal3 molecules to undergo self-association via N-terminal domains forming ligand-attached hetero-oligomers, permitting multivalent interactions. This dynamic process of oligomerization is influenced by types of CRD ligand and Gal3 concentration [133,135,136] and dependent on the presence of multiple proline residues (P50/51/55/60/64/67) at the N-terminal domain [137]. Cleavage of the N-terminal domain that contains repeats of collagen-like motifs largely prevents its function despite the CRD ligand binding remaining intact [133,138]. Thus, the N-terminal domain and oligomerization are essential for the full function of Gal3. Earlier studies indicated formation of heterogeneous crosslinked oligomers, mostly Gal3 pentamers and dimers [134]. However, a recent study on the involvement of Gal3-α5β1 integrin in endocytosis revealed the formation of stable tetramers, dimers and trimers, rather than pentamers [139]. Whether this occurs under different experimental conditions, e.g., nature of ligand and cell types, remains to be studied. Such structural oligomerization enables higher Gal3 capacity and stability of ligand binding (Figure 2B) critical for its pleiotropic actions in modifying activities of a wide range of proteins or membrane lipids. Both C-terminal and N-terminal domains are essential for its role in signal transduction, cellular adhesion, and lattice formation. Under diseased conditions, other molecules exert regulatory roles in Gal3 oligomerization. For instance, endotoxin LPS was found to interact directly with Gal3 at N- and/or C-terminals with subsequent enhancement of Gal3 oligomerization and activation of neutrophils [140].

There has been increasing interest in liquid phase biomolecular condensation as a newly recognized mechanism by which a cell can self-assemble and compartmentalize subcellular structures as liquid droplets without lipid membrane binding. This dynamic process is referred as liquid–liquid phase separation (LLPS) [141]. These intracellular liquid droplets can merge into larger condensates upon contact with each other. Gal3 protein is found to localize in macromolecular assemblies through LLPS [137,142,143,144,145]. Research on proteins involved in LLPS suggests that Gal3 possesses all required structural properties for its presence in biomolecular condensates, i.e., containing an intrinsically disordered N-terminal domain, undergoing oligomerization via multivalent binding, and extensive association with glycoconjugates [141,142,143,144]. Studies have shown that Gal3 participates in LLPS occurring in the cytosol, nucleus or plasma membrane [141,142,146], in which a proline-rich N-terminal tail plays a critical role [137,144]. The recognition of the role of Gal3 in LLPS represents an important evolution in research on Gal3, which opens an avenue for exploring novel Gal3 biology under physiological and pathological conditions. Zhang et al. recently reported that LLPS triggered by Gal3-α5β1 integrin crosslinking is critical for skin wound healing and that interruption of this mechanism by diabetes-induced accumulation of AGEs impairs wound healing [146].

The Gal3–glycoconjugate lattice is a multivalent crosslinkage on the cell surface membrane or membrane of intracellular organelles such as mitochondria, lipid droplets, endoplasmic reticulum (ER), Golgi bodies, and even nucleus (Figure 2C) [6,147]. Lattices incorporate Gal3–glycoconjugates into membrane proximal networks forming stable membrane domains or distinct transport complexes. Gal3–glycoconjugate lattices provide a good example of the ability of functional multivalency to strengthen weak interactions, such as protein–carbohydrate binding, through a wide assortment of scaffolds of Gal3 and carbohydrates (Figure 2C). This structural feature of Gal3 in oligomers is critical for its extracellular functions such as signal transduction, cell–cell interaction, cell–matrix interaction, cellular migration, receptor turnover, protein clustering or formation of caveolae, in which the presence of Gal3–glycoconjugate lattices is important [145,148]. The lattice structure also plays a key regulatory role in transmembrane transportation of proteins [145]. Studies have shown that the binding of Gal3 to glycoligands and formation of lattices are weakened by a low pH [149]. While the Gal3–glycoconjugate lattice is regarded as critical for cellular functionality, our current understanding of Gal3 lattices remains very limited. It is anticipated that the rapid progress in proteasome and glycoproteomics would reveal new information on the biological, pathological and therapeutic significance of Gal3 lattices.

### 3.4. Post-Translational Modifications of Gal3

The function of Gal3 is dependent on its CRD binding to glycoconjutates, oligomerization via the N-terminal domain and protein–protein interactions. Hence, PTMs of the Gal3 molecule such as proteolytic cleavage, multivalency or phosphorylation are expected to influence its function.

Post-translational glycosylation (O-GlcNAcylation). O-linked β-N-acetylglucosamine (O-GlcNAcylation) is a ubiquitous type of PTM that involves the addition of a single N-acetylglucosamine sugar onto a serine or threonine residue of a protein. In cancer cells or cells subjected to stress stimuli, intracellular proteins with O-GlcNAc represent a powerful signaling pathway that functions as an adaptive mechanism for cytoprotection. Lariwala et al. compared the levels of galectin O-GlcNAcylation and the abundance of galectins in liver tissue in squirrels in the winter and the summer and found that the levels of galectin O-GlcNAcylation and galectin protein expression were markedly lower in hyper-metabolic (summer) relative to hypo-metabolic (winter) status [150]. These changes were not accompanied by any differences in the expression of genes encoding enzymes of the O-GlcNAc cycle or galectins (including *Lgals3*). Thus, the stability of intracellular galectins may be regulated by galectin O-GlcNAcylation, which is important considering the distinct functions of Gal3 outside and within cells. Indeed, expression of *Lgals3* in cancer cells is enhanced following Gal3 O-GlcNacylation [109]. Moreover, secretion of Gal3 is regulated by its O-GlcNacylation, by which Gal3 senses nutrient information, represented by cell surface glycosylation, and intracellular O-GlcNAcylation with corresponding alteration of its biological function [151].

Ubiquitination. Studies have revealed Gal3 ubiquitination degradation in cancer cells, where upregulated Gal3 is observed in many cell types and associated with poor prognosis [18,152,153]. In cancer or non-cancer cells, several studies have observed Gal3 ubiquitination by a class of ubiquitin E3 ligase tripartite motif-containing proteins (TRIM16/21/29/49) [154,155,156,157]. Another E3 ligase, RNF8, was found to mediate Gal3 degradation via the ubiquitin–proteasome machinery [158]. Fang et al. reported in human hepatocellular carcinoma that *LGALS3* stability is increased owing to the upregulated ubiquitination-specific protease15 (USP15) that mediates Gal3 deubiquitination modification, with resultant enhancement of hepatocellular carcinoma stemness, proliferation and chemotherapy resistance [159]. In CD8+ T-lymphocytes, ubiquitination-mediated degradation of Gal3 is enhanced following inactivation of ERK1/2, which is accompanied by reduced release of Gal3 [160]. Further study is required to test Gal3 ubiquitination and its biological consequences in cardiovascular tissues.

Phosphorylation. Gal3 phosphorylation has been shown to influence its secretion, distribution, degradation and oligomerization. There are multiple sites within Gal3 molecules where phosphorylation takes place on serine or tyrosine residues (Figure 2B), including Ser-6 and Ser-12 within the N-terminal peptide and several tandem collagen-like repeats containing Tyr residues (Tyr-79, -107 and -118) in the internal repeating domain. Gal3 is phosphorylated in Tyr residues by kinases including c-Abl (a kinase localized in the cytoplasm and nucleus) and casein kinase 1 (CK1). Phosphorylation at Ser residues is mediated by CK1 or glycogen synthase kinase-3β (GSK-3β) (Figure 2B) [161,162,163,164]. Serine and tyrosine phosphorylation of the Gal3 molecule by CK1 or c-AbI facilities its entering into lysosomes and subsequent cleavage/degradation [163,164,165,166], which promote cancer cell death [166,167], reduce its capacity for ligand binding via CRD, and limit its nuclear exporting [164,168,169]. Serine phosphorylation by GSK-3β could regulate its localization and its participating in signal transduction like the Wnt-β-catenin pathway [161]. In cancer cells, the well-documented action of Gal3 in preventing programmed cell death (apoptosis, anoikis) is lost following N-terminal Ser-6 phosphorylation together with the attenuated ligand binding [166]. Ser-6 phosphorylation of Gal3 is also critical for its nuclear entry and accumulation (see below) and influences cellular translocation. Studies have shown that the ligand-binding capacity of Gal3 is markedly reduced following phosphorylation at various sites (Ser-6, Tyr-107, Tyr-118) [162,166,168], while Gal3 Tyr-107 phosphorylation blocks Gal3 cleavage [170]. Gal3 phosphorylation can be reversed by protein dephosphotase type-1 [164]. While the nuclear fraction of Gal3 contains both phosphorylated and non-phosphorylated isoforms of Gal3, the nuclear export fraction of Gal3 contains exclusively the phosphorylated isoform [169,171]. Accordingly, cleavage or phosphorylation plays an important role in regulating Gal3 function via altering its functional multivalence, localization and interaction with glycoconjugates [162,163,164,166,172]. Thus, distribution, stability and multiple biological activities of Gal3 are regulated by its phosphorylation. Future investigation on Gal3 phosphorylation under diseased settings would enrich our understanding of Gal3 biology and potential therapeutic implication.

Cleavage with altered functional multivalency. Following the first study by Ochieng et al. on MMP-mediated cleavage of Gal3 by in breast cancer [173], it has been well documented that MMPs (MMP-2, -9, -12) and membrane type-1 MMP (MT1-MMP) are able to cleave Gal3 at its extracellular collagen-like domain, generating intact CRD and N-terminal peptides of varying lengths that retain lectin-binding activity but lose capability of multivalence (Figure 2B) [163,174,175]. MMP-mediated cleavage of Gal3 is enhanced by overexpression of MMPs but inhibited by TIMP-2 [163,173]. By using specific antibodies recognizing the cleaved and non-cleaved forms, in situ zymography and point mutation, MMP-2, MMP-9 and MT1-MMP are found to cleave Gal3 at amino residuals of collagen-like domain of Ala-32−Try-63 peptide (Figure 2B) [174,175,176]. Whereas Gal3 is commonly regarded as a cellular marker and a chemotactic molecule for macrophages, atherosclerotic plaques contain Gal3-positive macrophages (M1) as well as a subtype of Gal3-negative macrophages, and both cell types determine plaque composition and stability [73]. When Gal3 is cleaved by MMP-12 into 22 kDa fragments containing the entire CRD and a 9 kDa N-terminal peptide, there is worsening of advanced plaque phenotype. Indeed, there are clinical reports showing that patients with premature myocardial infarction had a high circulating level of soluble Gal3, likely contributing to the instability of coronary atherosclerotic plaques with increased risk of ischemic events [177]. Relevant to Gal3 cleavage by MMPs are findings on cancer cells showing that Gal3 is able to upregulate expression of MMP-1, MMP-2 or TM1-MMP [174,178]. Gal3 can also be cleaved by prostate-specific antigen (PSA, a chymotyrosin-like serine protease) at the site after Tyr-107, resulting in Gal3 being largely a monomer, owing to the loss of its multivalency [170].

## 4. Intracellular Trafficking, Secretion and Quaternary or Gal3 Lattices

After being synthesized in the cytoplasm, Gal3 undergoes dynamic processes of intracellular trafficking, sorting, extracellular secretion or re-entering into cells. Through its multivalent property, Gal3 can also form complex quaternary structures intracellularly by interacting with its glycoconjugates or even form complex quaternary structures or lattice meshwork over the surface of organelles or cellular membrane. Given the efficient renal clearance of circulating Gal3, the extracellular secretion of Gal3 is a major determinant for the circulating level of Gal3.

### 4.1. Intracellular Trafficking and Extracellular Exporting of Gal3

Studies have shown that intracellular Gal3 mainly distributes in endosomes or vesicles [179] and that Gal3 content in exosomes increases upon pathological stimuli such as inflammation or oxidative stress [27]. Proteomic analysis identified the presence of Gal3 in vesicles or exosomes prepared from a range of tissues, blood, body fluids or cell culture supernatant [180,181]. Emerging studies have shown elevated exosome Gal3 levels as a biomarker for cardiovascular or other diseases [27,182]. Further study on the mechanisms controlling the secretion of Gal3 would improve our understanding of Gal3 as a biomarker and a therapeutic target.

Protein secretion occurs through conventional and unconventional pathways [183]. The conventional secretory pathway is through the ER/Golgi apparatus for cytoplasmic proteins that contain a signal peptide or signal sequence that is usually composed at the N-terminus of 16–30 amino acids and guides the protein localization. During the transportation through the conventional ER/Golgi secretory pathway, the signal sequence is cleaved by ER membrane proteins of the signal peptidase complex (SPC) prior to secretion. Gal3 does not contain a signal sequence and therefore cannot traverse the ER/Golgi apparatus for secretion after being synthetized in cytoplasmic ribosomes. However, Gal3 can be secreted through non-conventional mechanisms to the surface plasma membrane and extracellular environment. There is also evidence that Gal3 penetrates the lipid bilayer bidirectionally through interacting with membrane glycolipids (sphingolipids and phospholipids as structural lipids) via its CRD binding [184]. Gal3 secretion starts from recruitment of cytoplasmic Gal3 into intraluminal vesicles as transporting vehicles and then proceeds to release in the form of exosomes. This process of sorting, intracellular transporting and exosomal secretion of Gal3 depends on the system of endosomal sorting complexes required for transport (ESCRTs), in which several components of the system (e.g., Tsg101 and Vps4a) interact with certain motifs of Gal3 molecules [181]. Amino acid mutations in either of these components in ESCRTs or Gal3 abolished the intracellular Gal3 accumulation in intraluminal vesicles and exosomal secretion. The presence of Gal3 in vesicles involves Gal3 binding via CRD to glycoproteins. Vesicular Gal3 may then regulate intra- or extracellular locations of vesicles as well as vesicle lysis with the release of vesicular contents including Gal3 per se [180]. Enhanced synthesis and secretion of Gal3 occur in tumor cells, promoting tumor growth and metastasis [185], or in residential macrophages, facilitating inflammatory and fibrotic signaling [186].

The membrane-covered transport cargos, including vesicles and endosomes, can shuttle between intracellular compartments and between cells or tissues if released into the extracellular milieu. These vesicles are composed of various lipids, nucleic acids and proteins. After being secreted, Gal3 may enter back into cells by endocytosis. This process is dependent on multiple mechanisms, including the clathrin-mediated endocytosis or alternative pathways involving caveolin, flotillin, phagocytosis or micropinocytosis. Gal3 re-entry is critical for the turnover of extracellular Gal3 and for its regulation of various cellular functions. Interestingly, Lepur et al. found distinct mechanisms in Gal3 endocytosis between M1 and M2 macrophages. In THP-1-derived M1 cells, Gal3 endocytosis can be blocked by chlorpromazine, a blocker of clathrin-mediated endocytosis, or by mutations of the Gal3 N-terminal domain. In M2 macrophages, however, Gal3 endocytosis is CRD-dependent albeit the presence of other domains is also required [187]. The demonstration of the distinct mechanism in Gal3 endocytosis in different cell types implicates distinct cellular functions that require involvement of Gal3. Importantly, endocytosis of membrane receptors (e.g., GPCRs, VEGFR), carrier proteins, signaling molecules or even metabolites (e.g., hexosamine) occurs together with Gal3 [25,188,189,190] or is regulated by membrane Gal3 lattices [145]. Thus, the presence of Gal3 (and other lectins) and Gal3–glycoconjugate interaction are critical for biological processes that involve turnover of membrane vesicles for exocytosis and endocytosis, as well as intracellular events, including formation of autophagosomes and phagocytosis.

### 4.2. Factors Regulating Gal3 Secretion

The processes of intracellular transportation and extracellular secretion of Gal3 are influenced by its phosphorylation. In mouse embryonic fibroblasts, the secretory process of Gal3 is dependent on its tyrosine phosphorylation that requires calpain small subunit 4 (Calpain 4, a Ca^2+^-dependent protease) [165]. Both Gal3 secretion and Calpain 4 jointly regulate cell migration [191]. Gal3 entering vesicles or endosomes is pH-dependent, requires cargo proteins (e.g., β-integrin or CD44) and glycophospholipids, and is regulated by galectin lattice formation [180]. An early study showed that, in activated macrophages, about one third of Gal3 appears on the cell surface, and that secretion of Gal3 is stimulated by increased intracellular Ca^2+^ level [192].

Studies have discovered that Gal3 secretion might be disproportional to its expression level. Cells differ widely in their capacity of Gal3 secretion ranging from 1% to 45% of the cellular Gal3 pool [165,185,192]. In reparative macrophages prepared from the infarct heart, treatment with IL-10 induced pronounced intracellular Gal3 expression without simultaneous augment of extracellular secretion [108]. There have been contradictory reports whether it is the intracellular expression or extracellular secretion of Gal3 that is involved in the phagocytic clearance of apoptotic cells by macrophages [193,194]. In the Mst1-cTG mouse model of DCM, high intracellular Gal3 content in cardiomyocytes (Figure 5) is not accompanied by detectable increments in blood levels of Gal3 [32,91,104], suggesting that Gal3 release by cardiomyocytes of Mst1-cTG mice is negligible. Thus, these findings cast doubts on the role of extracellular Gal3, either secreted or exogenously administered, in mediating Gal3 actions, including immune cell activation, transdifferentiation and activation of fibroblasts. We observed that myocardial βAR signaling promotes Gal3 secretion by cardiac cells into peripheral circulation. Transcardiac gradients of Gal3, analyzed by simultaneous blood sampling from peripheral veins and the coronary sinus, were clearly present in mice with ischemia–reperfusion or with enhanced cardiac βAR activity [104]. In mice administered with β-agonist isoproterenol, both cardiac and blood levels of Gal3 increased and this increment is more pronounced in Mst1-cTG compared with wild-type controls, suggesting mobilization of Gal3 from the large intracellular pool of cardiomyocytes in Mst1-cTG mice [104]. This effect of isoproterenol can be partially inhibited by β_1_- and β_2_-blockers, confirming the involvement of both β_1_AR and β_2_AR subtypes in mediating cardiac secretion of Gal3 [31,104]. It is likely that the sudden increase in myocardial mechanotransduction, evoked by the inotropic and chronotropic actions following βAR activation, is responsible for a rapid Gal3 release in the Mst1-cTG model. Indeed, Kwon et al. reported that stimulation of mice with α- and β-adrenergic agonists differentially altered the contents of circulating extravesicular vesicles [195]. In cultured cancer cells that were in a near-confluent and spreading state, Baptiste et al. found that, whereas Gal3 release into culture medium was extremely low, the release process could be promptly activated and sustained by stimuli like cell detachment that involves a mechano-sensing mechanism [196]. In monocytes, stimulation with GM-CSF promotes secretion of Gal3 into the medium in a significant amount, which is repressed by IL-4 [111].

### 4.3. Signaling Regulation of Membrane Receptors or Integrins by Gal3

**Figure 6:**
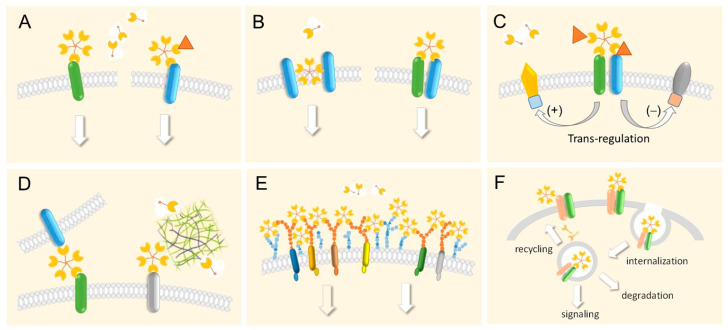
Modes of regulation by Gal3 of membrane receptor signaling. (**A**) For receptors with activation requiring receptor dimerization or assembly with other partner proteins or co-receptors like integrin, oligomerized Gal3 binding facilitates assembly of the receptor signaling complex; (**B**) Acting as a non-canonical ligand, Gal3 binds to and regulates receptor downstream signaling. Gal3 can also simultaneously bind to receptor and its canonical ligands to assemble receptor–ligand complex with facilitated signaling. (**C**) Gal3 participates in assembly of receptor and partner proteins with transregulation of other membrane receptors. (**D**) Gal3 plays a pivotal role in facilitating receptor–receptor- or receptor–ligand-mediated cell–cell or cell–matrix interaction through binding to these receptors, integrin or extracellular matrix; (**E**) Through lattice structure to hold signaling proteins in close proximity, Gal3 facilitates formation of clusters of membrane receptors, ligands, co-signaling protein and kinases; (**F**) Gal3 regulates membrane receptor turnover, thereby influencing receptor localization and activity.

Membrane receptor signaling. The extracellular and membrane Gal3 plays a key role in mediating its pleiotropic actions through membrane receptors and intracellular signaling pathways [10,13,14]. Current research findings indicate several working modes by which Gal3 acts as a central signal integrator regulating the activity of numerous membrane receptors and their downstream signaling cascades. As illustrated in Figure 6, acting as a non-canonical ligand, Gal3 binds to receptors, mediating activation or suppression of downstream signaling (Figure 6A). There are reports on activation of receptor tyrosine kinase (RTK) [197], VEGFR, activation via platelet glycoprotein-VI (GPVI) [198], CD45 and CD98 of T-cells [199,200], as well as inactivation of inulin receptor by Gal3 through this mechanism [201]. Gal3 can also bind to a receptor or its canonical ligands to assemble the receptor–ligand complex with facilitated signaling (Figure 6B), as observed for TGFβ/TGFβR, LPS/TLR, danger-associated molecular patterns (DAMPs)/TLRs, advanced glycation end-products (AGEs)/RAGEs/AGERs, cytokines or chemokines and their receptors [66,202,203]. There is evidence that Gal3 and chemokines form heterodimers, thereby altering inflammatory signaling via specific receptors [202,204]. For receptors like VEGFR and TLR4, their activation requiring receptor dimerization or assembly with other partner proteins or co-receptors like integrin, binding by Gal3 oligomers facilitates the assembly of the receptor signaling complex (Figure 6C) [64,80,83,86,146,205,206,207]. Gal3 plays a pivotal role in facilitating cell–cell or cell–matrix interactions through its joint binding to receptors, integrin or ECM proteins involved (Figure 6D) [208,209]. Alternatively, Gal3 participates in the assembly of receptor and partner proteins leading to transactivation of other membrane receptors (e.g., VEGFR-integrin) [210,211]. Through formation of lattices, Gal3 involves clustering of signaling complexes composed of ligands, receptors and signaling molecules (Figure 6E) [148,206,212,213]. Furthermore, Gal3 regulates the processes of membrane receptor internalization and recycling, thereby influencing receptor localization and activity (Figure 6F) [145,187,188]. Collectively, Gal3-mediated regulation of signaling through membrane receptors constitutes a pivotal mechanism of its biological actions.

Integrin signaling. Integrins are a large family of heterodimer transmembrane adhesion and signaling proteins that regulate a range of biological processes involving cell–cell and cell–ECM attachment and bidirectional signal transmission [214,215]. Being glycoproteins, integrins, particularly β1-integrin, have been found to extensively interact with Gal3 in different modes. First, integrins exhibit high affinity to Gal3 and can form integrin–Gal3 clusters [216], and together with glycolipids, oligomeric Gal3–integrins further form lattices at the cell surface membrane [217]. The direct Gal3–integrin interaction is important for wound healing and angiogenesis, and such actions can be attenuated under conditions of diabetes where regional accumulation of AGEs competes for Gal3 binding, thereby weakening Gal3–integrin interaction [146]. Gal3–integrin accumulation also restricts mobilization of membrane receptors like EGFR, and formation of the Gal3/integrin-linked kinase (ILK)/Src complex mediates activation of caveolin-1 and RhoA by phosphorylation [67,148,205,211,218]. This pathway is critical in controlling actin organization and cell migration. Second, there exist mutual interactions of Gal3 and integrins. With the aid of caveolin-1, Gal3 binds to integrins and kinases (e.g., focal adhesion kinase, FAK, and phosphatidylinositol 3-kinase, PI3K) to induce integrin activation. β1-integrin signaling promotes the expression of Gal3 likely via NF-κB, Stat3, YAP or HIF-1α signaling pathways [67]. However, Gal3 is also involved in internalization of β1-integrin, thereby inhibiting the formation of adhesions. In cancer cells, Gal3 deletion reduced content of integrins at the plasma membrane by accelerating integrin internalization [216]. In cancer or brain tissues, Gal3 inhibits the expression of β3-integrin and facilitates integrin internalization, thereby diminishing membrane signaling and formation of integrin–actin cytoskeleton organization [152,172,216]. Third, Gal3–integrin interaction regulates membrane receptor signaling of adjacent cells. Gal3 recruits active integrins and enhances the formation of cell–cell or cell–ECM adhesions by altering receptor clustering [23,205,209,212]. On the other hand, Gal3 regulates endothelial cell motility and angiogenesis by binding to α1β1-integrin or forming a complex with α2β1-integrin and neuron/glial antigen 2 (NG2, a membrane protein critical for cell adhesion, migration and signaling) [219]. McDonald et al. found that EGFR triggers internalization of integrins and other glycoproteins through endocytosis that is driven by Gal3–glycoprotein binding [220]. Integrins closely participate in EGFR signaling, which is facilitated by the formation of the Gal3/EGFR/Akt/FOXO3 signaling complex [221,222,223]. Likewise, in cancer tissues, coupling of FAK and integrins by Gal3 oligomers is critical in mediating transactivation of the VEGF-VERFR/kinase insert domain receptor (KDR)/fetal liver kinase (FLK) and FGF-FGFR signaling pathways, ultimately enhancing angiogenesis [25,210,224,225].

## 5. Intracellular Localization, Transportation and Actions of Gal3

**Figure 7:**
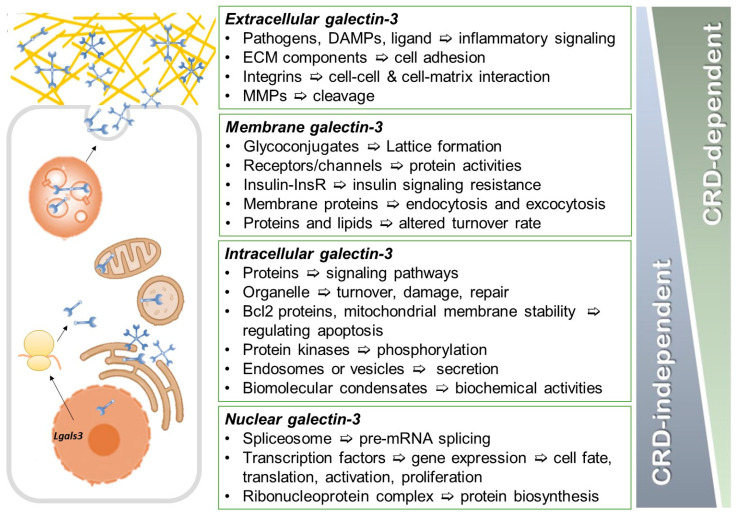
Localization-dependent binding partners and biological actions of Gal3. Apart from extracellular activities of Gal3 that are mediated through CRD binding to glycoconjugates, through protein–protein interactions, Gal3 also plays important roles in the intracellular compartments, where Gal3 influences cell signaling by interacting with signaling proteins in the cytoplasm, and RNA splicing through binding to components of spliceosome complex, mitochondrial function, or gene expression by interacting with nuclear transcription factors.

The biological functions of Gal3 are tightly dependent on its extracellular or intracellular localization and can be CRD-dependent or -independent (Figure 7). Several extensive review articles have addressed Gal3–glycoconjugate interaction via CRD, through which membrane or cytoplasm Gal3 regulates numerous signaling pathways, gene expression and biological actions in various cell types [6,13,197,226,227]. Therefore, our discussion here will concentrate on transcriptional regulation of its target genes mediated via nuclear Gal3 to explore its CRD-independent actions more. This issue is important since it may explain some controversial findings like differential efficacy of CRD-based Gal3 inhibitors versus *Lgals3* gene deletion or intracellular versus extracellular Gal3.

### 5.1. Nuclear Localization and Mechanism of Cytoplasmic–Nuclear Gal3 Trafficking

After being synthetized in the cytoplasm, Gal3 undergoes dynamical transportation, either being externalized into the extracellular space, penetrating the plasma membrane, or translocating into the nucleus and other organelles. There is evidence that Gal3 distribution is cell-type-dependent [176]. High levels of nuclear Gal3 have been reported in various cancer cell types [68,228,229,230]. In CVD, robust upregulation of Gal3 occurs in infiltrated macrophages whereas, in fibroblasts or cardiomyocytes in heart disease like myocardial infarction or cardiomyopathy [31,51], nuclear localization of Gal3 in relevant cell types, in particular cardiomyocytes, remains inconclusive. In a DCM model (Mst1-cTG) with pronounced upregulation of Gal3 in cardiomyocytes, we recently found by immunohistochemistry or immunoblotting analyses a high content of nuclear Gal3 in the myocardium of DCM versus non-TG mice (Figure 5A–C) [131].

**Figure 8:**
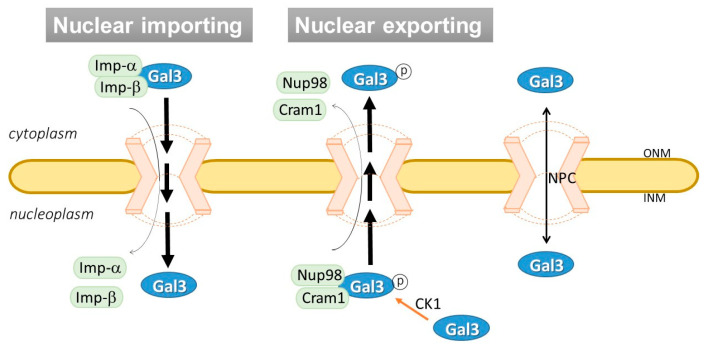
The machinery that mediates nuclear importing and exporting of Gal3. Abbreviations: NPC: nuclear pore complex; Imp: importin; Cram1: arginine methyltransferase; CK1: casein kinase 1; Nup: nucleoporin. ONM and INM indicate outer- or inner-nuclear membrane. ⓟ: phosphate group.

Nuclear importing and exporting of Gal3 require a complex transportation machinery through a membrane gateway called the nuclear pore complex (NPC), which is a large macromolecular structure composed of about 30 different proteins termed nucleoporins (Figure 8). The NPC spans phospholipid bilayers of the nuclear membrane with a pore size of about 120 nm. This complex forms the only gateway for selective and bidirectional transportation of nucleocytoplasmic proteins. Small molecules may pass through NPC pores by diffusion, while transportation of large proteins is achieved by selective binding to nuclear transport receptors, termed karyopherins, for import (importins) or export (exportins). Usually, the transport process requires karyopherin binding to target proteins in the cytoplasm at the site of the nuclear localization signal (NLS). The nuclear importing of Gal3 in the form of a monomer (26–30 kDa) can be mediated by passive diffusion through the NPC, but active transport is essential for Gal3 in the form of oligomers (Figure 8) [176]. Whereas nuclear importing may also occur independent of CRD [19], Gal3 nuclear transportation via the NPC requires the presence of the C-terminal domain sequence acting as a NLS, and deletion of the C-terminal domain of the Gal3 molecule prevents its nuclear localization [231,232]. Specifically, the last 28 amino acids of the C-terminal region of Gal3 play an indispensable role in determining its intracellular localization. The importin-α/β complex is involved in Gal3 binding and nuclear import, while Nup98 mediates Gal3 export into the cytoplasm through binding to CRD. Gal3 is known to be phosphorylated at the residue of N-terminal Ser-6 by CK1 [233], which impacts its localization. Whereas the phosphorylated form of Gal3 exists in both cytosolic and nuclear fractions, the non-phosphorylated Gal3 presents exclusively in the nucleus, indicating that phosphorylation is required for Gal3 nuclear-to-cytoplasm transportation [171]. In cancer cells, hypoxia exposure induced a marked upregulation of *Lgals3*/Gal3 at mRNA and protein levels, which was accompanied by redistribution of Gal3 from the nucleus to cytoplasm and membrane [68]. Such hypoxia-stress-evoked Gal3 translocation may imply cellular self-protection through enhanced anti-apoptotic action of cytoplasmic Gal3. It should be noted that the research on Gal3 shuttling has largely been conducted in cancer cells, and the application of this body of knowledge on cardiovascular cells, particularly non-proliferative cardiomyocytes, requires further investigation.

### 5.2. Nuclear Actions of Gal3 in Regulating Expression of Genes

Nuclear Gal3 has two primary functions, pre-RNA splicing and transportation and transcriptional regulation of target genes.

Pre-mRNA splicing via Gal3–spliceosome interaction. Classical RNA-binding proteins influence the fate of mRNA molecules at multiple levels, including splicing, nuclear export, storage, stability and protein translation. Acting as a non-classic RNA-binding protein owing to the absence of a RNA-recognition motif, Gal3 is crucial for pre-mRNA splicing. Through its CRD and also N-terminal domain (via PYG-rich repeats) (Figure 3A), Gal3 incorporates into a multiprotein complex, termed a spliceosome, that forms a complex with the U1 small nuclear ribonucleroprotein-snRNA [141], although the binding of Gal3 with the U1 small nuclear RNA (snRNP) is weak relative to that with more mature spliceosomal pre-mRNA. Nuclear Gal3 functions as a splicing factor of unprocessed heterogeneous nuclear RNAs (hnRNA), or pre-RNA. Coppin et al. showed that, despite the absence of an RNA-recognition motif, Gal3 is able to interact with heterogeneous nuclear ribonucleoprotein and indirectly with pre-mRNAs in the nucleus, regulating the splicing process and stability of mRNAs [234,235]. Research has implicated direct or indirect interactions of Gal3 with pre-RNAs in the nucleus or mature spliced mRNA in the peri-nuclear region or the cytoplasm, by which Gal3 post-transcriptionally regulates mRNA through simultaneous interaction with the spliceosome. Apart from the pre-mRNA-splicing function of Gal3, there is no report to indicate that Gal3 plays a role in other steps of mRNA metabolism/maturation. Gal3 also interacts with ncRNA in the nucleus [116]. Recent studies have demonstrated glycosylation modification of RNA (glycoRNA) [236,237], and it remains to be studied whether Gal3 directly interacts with glycoRNAs.

Transcriptional regulation of target genes by intracellular Gal3. Largely based on cancer research, it has become clear that Gal3 achieves its transcriptional regulation through several mechanisms. Studies in cancer or immune cells have found that Gal3 directly binds to numerous transcription factors, including AP-1, HIF-1α, heat shock factor-1 (HSF-1), early growth response1 (EGR1), WT-1, CREB, β-catenin, transcription factor 4 (TCF4), lymphoid enhancer-binding factor 1 (LEF1), cyclins, MRTF-A and NF-κB [40,57,238,239,240,241]. As a transcription co-regulator, such interaction between Gal3 and transcription factors influences the stability and also transcriptional activity of transcription factors, either activation or repression. Song et al. found that Gal3 is able to increase nuclear β-catenin content by preventing its degradation [46]. EGR1 plays a key role in cancer growth and metastasis and its stability is governed by the activity of the E3 ligase TRIM49. The EGR1–Gal3 complex leads to detachment of EGR1 from TRIM49 and hence an increased EGR1 stability. Such action can be reversed by restoration of TRIM49 expression or use of anti-Gal3 therapy [157]. In human breast epithelial cells, Lin et al. showed that nuclear Gal3 facilitates and stabilizes the formation of nuclear protein–DNA complexes, including transcription factors CREB and Sp1 at the cyclin D1 promoter with enhanced expression of cyclin D1 as well as activated cancer cell proliferation and metastasis [239].

Transcriptional regulation by intracellular Gal3 has been reported in cancer and non-cancer disorders [10,242]. A study on colon cancer cells revealed that intracellular Gal3 binds to and stabilizes heterogeneous nuclear ribonucleoprotein Q, thereby promoting cell proliferation [243]. Nucleolin is a major nuclear protein with multiple functions primarily in ribosome biogenesis, stabilization and transportation of RNA, and chromatin remodeling via its interaction with histones. In melanoma cells, Gal3 interacts with nucleolin in the nucleus and cytoplasm with enhanced ribosome biosynthesis and chromatin reorganization [244]. The Gal3 lattices play a key role in the regulation of multiple processes, including inflammatory activation, transmembrane signaling and protein secretion [25]. In an animal model of acute renal injury with markedly increased expression of Gal3, Chen et al. reported that Gal3 directly binds to the 3’UTR region of the nuclear receptor-4a1 (Nr4a1) promoter, leading to upregulation of Nr4a1. Being a transcription factor, Nr4a1 mediates upregulation of BRCA1-associated protein 1 (Bap1) and facilitates ferroptosis with worsening of renal injury [245]. In another study on an animal model of acute renal injury, overexpressed Gal3 promotes histone lactylation at H3K18 with enhanced expression of fibroblast growth factor receptor 4 (FGFR4) via interaction with glycolytic pyruvate kinase M2 (PKM2), thereby facilitating renal fibrosis and the development of calcium oxalate stones [246]. As discussed in Section 4.3 (Figure 6), transcriptional regulation by Gal3 is also achieved by its regulation of numerous signaling pathways either through direct interaction with membrane receptors or signaling molecules and kinases. Lastly, nuclear Gal3 participates in nucleus–cytoplasm mRNA shuttling. Collectively, multiple nuclear and extranuclear actions of Gal3 converge on its transcriptional regulation.

### 5.3. Regulation of Cell Fate by Gal3

Cell proliferation. The proliferative property of Gal3 in cancer cells or other cell types, such as endothelial cells and fibroblasts, has been well documented. Research in cancer tissues or cells highlights a central role of nuclear Gal3 in regulating cell proliferation through interactions with numerous transcriptional factors that activate expression of cyclin A/D, p21, p27, c-Myc, CREB/SP1 (a cyclin expression promotor), Axin/β-catenin/GSK3β/transcription factor-4 (Tcf-4), or thyroid-specific transcription factor (TTF-1) (Figure 8) [19]. Interestingly, the proliferative action of Gal3 is in part attributable to its interference in the stability of p21, a tumor suppressor and key cell-cycle negative regulator [47]. Conversely, the expression of *Lgals3* is inhibited by p53 (Figure 3A) [47,72], the action contributing in part to the action of p53 as a tumor suppressor. Numerous studies either in cancer cells or proliferative cardiovascular cells have showed that Gal3 regulates signaling networks that govern cell fate, including stemness, proliferation and transdifferentiation. The signaling pathways involved include Jagged-1/Notch [210], β-catenin/Wnt/GSK2β [240,241,247,248], mTOR [249,250], integrin-RhoA-JNK signaling [251], CT-1 or LIF/JAK-STAT3-Gal3 axis [252], intermediate-conductance Ca^2+^-activated K^+^ channel (KCa3.1) [51,253], cyclin-D1/cAMP response element [239] or the YAP/FOXM/cyclin D1 axis [254]. The KCa3.1 channel promotes activation and proliferation of cancer cells, immune cells and fibroblasts likely through augmenting intracellular Ca^2+^ entry [253,255], and Gal3 promotes expression of KCa3.1 under diseased settings [51]. In cancer cells, AKT activity inhibits the oncogenic GSK-3β/β-catenin signaling and subsequent oncogene expression, and Gal3 enhances the GSK/β-catenin signaling by inhibiting AKT activity [240]. In the setting of organ fibrosis, Gal3 stimulates fibroblast-to-myofibroblast transition and myofibroblast proliferation [50,51,256,257,258,259]. Gal3 has been shown to promote the β-catenin/GSK3β signaling that drives endothelial-to-mesenchymal transition and pulmonary fibrosis [260].

Cell apoptosis. Gal3 plays key roles in cancer cell survival and the development of insensitivity to anti-cancer therapies through its direct interaction with well-known oncogenes like Bcl-2, Ras and Myc. In both in vitro and in vivo models of myocardial ischemia, the protection by *Lgals3*-KO against apoptosis was accompanied by reciprocal changes in Bax/Bcl2 expression at mRNA and protein levels [33]. Gal3 possesses the anti-apoptotic NWGR motif within the CRD, by which it binds directly to BH-1 Bcl-family proteins and regulates cell apoptosis (Figure 2B) [25,57,261]. Gal3–Bcl-2 binding stabilizes the mitochondrial membrane potential, thereby inhibiting release of cytochrome-*c* and subsequent cell apoptosis, which mediates tumor immune escape under conditions of anti-cancer chemotherapy or immunotherapy [20,22,43,44,45]. Other actions of Gal3, such as activation of oncogenes (Ras, c-Myc, Erk, p21), also contribute to its anti-apoptotic property. The Gal3–p21 interaction provides an explanation for the dual actions on cancer growth by p21, which acts primarily as a tumor cell repressor through cell-cycle arrest but can also exhibit anti-apoptotic action. Conversely, there also exists reciprocal inhibitory regulations between the tumor suppressor p53 and Gal3, hence the pro-apoptotic action of p53 is achieved in a large part through downregulation and inactivation of Gal3 [72,262]. Unlike the pro-survival action of Gal3 in cancer cells, numerous studies on heart disease have provided strong evidence for the pro-apoptotic action of Gal3 expressed in cardiomyocytes under conditions like myocardial ischemia [33,122,263,264] and various types of cardiomyopathy or cardiac senescence [265,266,267,268,269]. In cultured adult cardiomyocytes, overexpression of Gal3 induces a 5-fold increase in cell apoptosis [261]. Some studies in cardiac ischemia or infarct models demonstrated anti-apoptotic effect by *Lgals3*-KO [265], albeit loss of Gal3 interfered with the post-infarct fibrotic healing [97,259]. In Dahl salt-sensitive hypertensive rats, knockdown of Gal3 by the lentivirus shRNA technique reduced cardiomyocyte apoptosis and improved survival [252]. In the model of aortic aneurysm, inhibition of Gal3 reduced VSMC apoptotic death in addition to suppression of inflammatory responses [41].

### 5.4. Regulation of Mitochondrial Metabolism

For several decades, it has been well known that cancer cells commonly exhibit both Gal3 upregulation and metabolic reprogramming. Studies have implicated a causal role of Gal3 in mediating metabolic reprogramming in cancer cells, involving alterations of glycolysis and mitochondrial metabolism [270,271]. In cancer cells, Gal3 overexpression is associated with upregulation of genes critical in anaerobic glycolysis (e.g., glucose uptake-1, GLUT1, hexokinase, phosphofructokinase and LDHA) and activation of HIF-1α, PI3K, RAS and ERK1/2 signaling, collectively promoting glucose uptake and aerobic glycolysis, i.e. the Warburg effect [270,271,272]. Mitochondrial homeostasis is critical for cancer growth albeit the oxidative phosphorylation is reduced. Upregulated Gal3 in cancer cells maintains mitochondrial homeostasis likely through interaction with key regulators such as AMPK and peroxisome proliferator-activated receptors (PPARs), as well as suppression of the mitochondrial apoptosis pathway [273]. Despite this recognition in cancer cells, limited studies have explored the contribution of Gal3 in cardiac metabolic remodeling [274]. Emerging studies have indicated regulation of metabolism by Gal3 in CVD. In a murine model of high-fat diet feeding, *Lgals3*-KO mice exhibited less elevation in fasting levels of glucose and insulin [275], PPARγ-mediated accumulation of white adipose tissue or hepatic steatosis [276,277], and cardiac lipotoxicity and mitochondria damage versus control mice [278]. A number of regulators for mitochondrial and lipid metabolism have been implicated to play key roles in regulating cardiac metabolism, including PPAR family members, insulin signaling, CD36 (a membrane DAMP receptor and fatty acid transporter), HIF-1α, βAR signaling and Hippo-YAP pathway [106,274,276]. Diabetic research has revealed that Gal3 contributes to the onset of cellular and systemic insulin resistance [201]. Gal3, especially derived from macrophages, interacts directly with insulin receptors and interferes with the insulin signaling pathway [201,276]. mTOR and STAT3 signaling cascades are implicated in regulating metabolism of glucose, amino acids and fatty acids [279,280]. In studies on obese mice, Gal3 administration induced insulin resistance and glucose intolerance, whereas loss of Gal3 by genetic or pharmacologic means improved insulin sensitivity [201]. In patients with type-2 diabetes, higher circulating levels of Gal3 are associated with the degree of insulin resistance [275,281]. Gal3 also contributes to metabolic reprogramming induced by inflammatory signaling. In an LPS-induced sepsis model, Gal3 binds to LPS and, acting as an LPS censor, interacts with lysosome-anchored Rag GTPase-regulator-mTORC1 signaling, thereby promoting expression of glycolysis-associated genes [249]. Although this finding was made in the setting of LPS stimulation, such enhanced inflammatory signaling would pertain to CVD and metabolic disorders where inflammation plays a critical role in disease progression. Indeed, in high-fat-diet-induced cardiomyopathy with fibrosis, hypertrophy and elevated ROS, treatment with a Gal3 inhibitor (N-acetyllactosamine, LacNAc) ameliorated these phenotypes [250].

**Figure 9:**
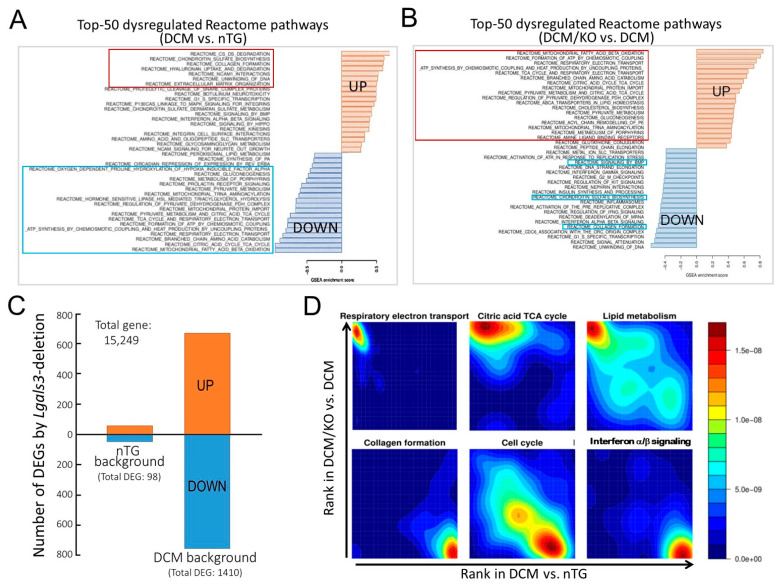
Transcriptional regulation of gene sets by Gal3 in a mouse model of dilated cardiomyopathy (DCM) due to cardiomyocyte-restricted Hippo pathway activation. The top-40 most significantly altered reactome gene sets in DCM relative to non-transgenic control mice (nTG) (**A**), or between *Lgans3*-deleted DCM mice relative to DCM mice (**B**). Note that the majority of downregulated gene sets are involved in mitochondrial metabolism ((**A**), blue rectangle), which were partially and significantly reversed in DCM/KO. Similarly, several profibrotic gene sets were upregulated in DCM mice (red rectangle in (**A**)) and were reversed in DCM/KO mice (blue rectangles in (**B**)). (**C**) Number of differentially expressed genes (DEG) by *Lgals3*-KO in wild-type (i.e., KO vs. nTG) and against DCM background (DCM/KO vs. DCM). MET: mesenchymal–epithelial transition; NCAM: neural cell adhesion molecule or CD56. (**D**) Rank–rank plot of expression of selected gene sets in these comparisons and the effect of *Lgals3* deletion against DCM background. The diagrams are replotted based on our original data published previously [32,91,131].

As discussed in Section 2.4, the Mst1-cTG DCM mice exhibited increased expression of Gal3 that accumulates in mitochondria (Figure 5C) [32], together with overt mitochondrial structural damage and metabolic abnormalities [91]. This model also exhibited a significant disturbance of metabolic regulatory signaling, indicated by downregulation of mitochondrial genes and PPAR family members (PPARδ, PPARα and PGC-1α), together with elevation of HIF-1α and glycolytic genes [91,282]. Using this model, we studied the effect of *Lgals3* gene deletion on myocardial metabolism by combination of the transcriptome and lipidome (Figure 9A,B) [283]. Whilst there are diverse and minor changes in myocardial lipid components and gene pathways in *Lgals3*-KO versus non-TG control mice, against the DCM background, *Lgals3*-KO (DCM/KO) is associated with a partial but significant reversal of DCM-induced changes in lipid classes and species, most notably an increase in sphingolipids, reduction in phospholipids or ether lipids, and reduction in triglycerides. In the DCM background, 9.2% of genes were differentially expressed (DEGs) by *Lgals3*-KO, whereas only 0.6% genes were DEGs for *Lgals3*-KO versus the non-TG group (Figure 9C). The transcriptome showed unchanged expression of gene sets of sphingolipid biosynthesis or the PPAR/PGC-1α family but revealed restoration of the downregulated pathways of mitochondrial metabolism in DCM/KO hearts (Figure 9A,B) [131]. Interestingly, *Lgals3* deletion in Mst1-cTG mice is associated with approximately 50% restoration of expression of about 1500 PPARα/PGC-1α target genes by the transcriptome, together with improved fatty acid metabolism by the lipidome, in addition to suppression of fibrotic genes (Figure 9C,D). Furthermore, upregulated expression of HIF-1α at both mRNA and protein levels, seen in this DCM model, was also reversed by *Lgals3*-KO [131]. These in vivo findings highlight, for the first time, the regulation of lipid and mitochondrial metabolism by upregulated Gal3 in cardiomyocytes, at least in part, through repression of mitochondrial metabolic genes. Regulation by Gal3 of lipid metabolism is also indicated by previous studies on cancer cells, immune cells and adipocytes [276,284], and Gal3 is found to augment inflammometabolic response under diseased conditions and promote lipid accumulation in human arteries [40]. Furthermore, Gal3 is able to bind to membrane structure and glycan-containing lipids such as sphingolipids [21,284,285,286,287], usually with adverse consequences under diseased conditions.

In addition to inducing metabolic dysregulation, mitochondrial Gal3 is implicated in suppressing mitochondria-mediated cell apoptosis through interaction with Bcl-2 proteins, regulation of cytochrome-*c* release or generation of mitochondrial ROS [43,273,288]. It remains unclear whether mitochondrial Gal3 might regulate mitochondrial turnover, energy metabolism and signaling function as well. Gal3 is known to prevent the process of mitochondria fission by limiting the recruitment of the regulator of fission protein DRP-1 [273]. Accordingly, loss of Gal3 in cancer or epithelial cells alters mitochondrial turnover reflected by accumulation of fragmented and round-shaped mitochondria. Gal3 localizes at the ER–mitochondrial interface, i.e., mitochondria-associated membranes (MAMs), and contributes to the integrative coupling of both organelles. The coupling of mitochondria with the ER is critical for cellular homeostasis and metabolism in settings of physiology and pathological stress, which may lead to accumulation of unfolded proteins [273]. In cancer cells or epithelial cells, Gal3 at MAMs preserves the integrity of mitochondrial network and MAMs, coordinates ER–mitochondria function, and modulates ER stress responses. Emerging evidence suggests the involvement of Gal3 in the functionality of MAMs and unfolded protein responses (UPRs) through interacting with MAM-localized proteins [273]. Whereas Gal3 does not alter the activity of the three UPR branches under basal conditions, following ER stress induced by thapsigargin Gal3 favors an adaptive UPR [273]. The effect of Gal3 in disease heart might be different, however. Under conditions of myocardial infarction, treatment with inhibitors against Gal3 (MCPs) or ER stress (4-phenylbutyric acid, 4-PBA) effectively reduced oxidative stress and fibrosis, suggesting that Gal3 in tandem with ER stress mediates post-infarct damage and healing [289].

## 6. Pro-Inflammatory and Pro-Fibrosis Properties and Gal3/Gal3-Binding Protein Interaction

Extracellular Gal3 plays a key role in the development of inflammation and fibrosis of CVD, which has been discussed in detail by several excellent reviews [6,10,13,14]. There is emerging evidence for extracellular Gal3 interacting with Gal3-binding protein (Gal3BP), contributing to inflammation and fibrosis. As discussed in the previous sections, intracellular Gal3 contributes significantly to these events through its regulation of relevant signaling pathways and transcription of numerous genes.

### 6.1. Pro-Inflammatory Action

NF-κB signaling plays a central role in inflammatory response by promoting expression of numerous inflammatory genes. There is strong evidence for Gal3 in facilitating NF-κB signaling by binding to either TLR4 or TLR4 ligands (Figure 4A) [64,80,86]. The INF-γ/JAK-STAT signaling pathway is also under regulation by Gal3. There are several mechanisms by which Gal3 promotes inflammatory signaling pathways and upregulates expression of inflammatory cytokines and chemokines (IL-1β, IL-6, IL-10/STAT3, TNF-α, iNOS, NOX4), or key signaling molecules (NF-κB, TLR4/MyD88/NF-κB, JAK/STAT3) [10,290]. In brain glial cells, exposure to exogenously added Gal3 induced rapid and sustained activation of the JAK-STAT-IFN-γ signaling cascade. This is achieved by tyrosine phosphorylation of key enzymes of this signaling pathway, such as STATs and JAK2, and also by upregulation of targeted inflammatory molecules [291]. There is also evidence for Gal3 in augmenting ROS generation or activation of NLRP3–inflammasome response [207,292,293,294,295]. Gal3 is able to activate subtypes of immune cells and regional build-up of Gal3 derived from inflammatory cells, in particular macrophages, forms a feedback loop promoting the inflammatory–fibrotic cascade in diseased settings such as myocardial infarction [296], viral myocarditis [297], hypertrophic cardiomyopathy [298], brain ischemia [299], renal failure [186], arteriosclerosis [73,300] and pulmonary hypertension [301]. Gal3 per se is able to directly bind to chemokine CXC receptors like a ligand, forming a heterodimer, an action that is CRD-independent [204]. In an acute renal injury model, release of Gal3 by injured cells exacerbates renal inflammation and damage through platelet activation via GPVI and subsequent formation of monocyte–platelet aggregates and activation of M1 macrophages [198]. Inflammation mediated by brain microglial cells is critical in the onset of Alzheimer’s disease. Liu et al. developed a Gal3 inhibitor, FJMU1887, by using AI-added structure design [302]. When tested in murine models of Alzheimer’s disease, FJMU1887 showed potent inhibition of brain inflammation and Aβ-plaque deposition. This drug was found to bind to and promote degradation of Gal3 protein, thereby disrupting the interaction between Gal3 and the triggering receptor expressed on myeloid cells 2 (TREM2), leading to diminished inflammatory signaling in microglial cells [302].

Notably, Gal3 also possesses immune suppressive action. For example, its pro-cancer action is, in a significant part, attributable to its suppression of anti-cancer immunity. A recent study reported that Gal3 derived from a subtype of breast cancer cells induces expansion of T regulatory cells and elevated levels of IL-10 and IL-35 with subsequent immunosuppression [200]. Furthermore, in CD8+ T-cells, Gal3 induces mitochondrial dysfunction through downregulating expression of mitochondrial oxidative genes with a resultant increase in ROS and exhaustion of T-killer cells [200]. The inflammasome pathway is crucial in innate immunity. Composed of multiple proteins like NLRP3, caspases and pro-cytokines, the inflammasome is formed and activated within the cytosol of immune or epithelial cells upon pathological stimuli. Gal3 plays a pivotal role in the priming and activation of NLRP3–inflammasome through direct binding to DAMPs and TLRs [207,293,294,303,304]. In several diseased models, including high-fat-diet-induced fatty liver disease [207], diabetes-associated atrial remodeling [304], doxorubicin-induced cardiotoxicity [303], acute colitis [294] or host response to bacterial infection [293], such action is attenuated by *Lgals3*-KO or Gal3 inhibitors, leading to amelioration of inflammatory responses.

Numerous studies have emphasized a pivotal role of macrophage-derived Gal3 in pathological conditions. Following myocardial infarction, Gal3-high macrophages play a key role in the clearance of dead cells by phagocytosis. In patients or mice with aortic aneurysm, there was a remarkable upregulation of Gal3 by M1 macrophages in aortic tissues contributing to VSMC apoptosis [41], and inhibition of Gal3 in mice reduced the risk of aneurysm, efficacy associated with downregulation of inflammatory factors and MMP-9. In type-1 diabetes, macrophage-secreted Gal3 suppresses differentiation and function of regulatory T-cells, which exacerbates pancreatic autoimmunity and diabetes [305]. Shirakawa et al. showed that activation of macrophages and expression of osteopotin are dependent on upregulation of Gal3 in M2 macrophages, which acts intracellularly to promote expression of genes like EGFR and reparative fibrotic response [108].

### 6.2. Pro-Fibrotic Action

The pro-fibrotic feature of Gal3 has been well documented by numerous studies in a variety of diseased conditions involving the heart [32,50,250,257,259,266,267], lungs [37,66,306], the kidney [307] and the liver [256,308]. Two mechanisms are responsible for the pro-fibrotic action of Gal3, i.e., promoting the expression of fibrotic genes and interacting with key fibrotic factors including osteopontin, TGF-β, platelet-derived growth factor (PDGF) and connective tissue growth factor (CTGF) [32,50,256,259,292,309]. Notably, the pro-inflammatory property of Gal3 also contributes to its pro-fibrotic action. Following myocardial infarction, a subclass of macrophages (CD206+) with high *Lgals3* expression showed exclusive upregulation of *Spp1* (gene encoding osteopontin), which is enhanced by IL-10. The synergistic signaling of IL-10/Gal3/osteopontin contributes to phagocytotic clearance of dead cells, an event preceding the subsequent fibrotic healing [108].

The pro-fibrotic action of Gal3 is attributable in a major part to its transcriptional regulation of fibrotic genes. In models of organ fibrosis, interventions including *Lgals3*-KO significantly repressed the fibrotic gene profile with downregulation of the expression of key fibrotic genes including TGF-1β and CTGF [14,32,103,259,309]. In Mst1-cTG mice with DCM and severe myocardial fibrosis, *Lgals3* gene deletion either partially (heterozygous) or completely (homozygous) mediated “dose-dependent” downregulation of numerous genes related to fibroblast activation, biosynthesis of ECM proteins and collagen modifications. These changes in gene expression are accompanied by approximately 40% reduction in tissue content of collagen, amelioration of ventricular dysfunction and improvement of the intracardiac conduction delay [32]. In rats subjected to MI, treatment with CRD-targeted Gal3 inhibitors inhibited excessive fibrotic healing in the border regions and improved ventricular performance [259]. Recent studies have revealed a pivotal role of K_Ca_3.1 channels in fibrogenesis [253]. K_Ca_3.1 is constitutively expressed in non-excitable fibroblasts, inflammatory cells and VSMCs and mediates cell activation through regulating membrane potential with enhanced Ca^2+^ influx. Upon pathological stimuli, K_Ca_3.1 channels are upregulated and activated in fibroblasts or macrophages, leading to cell activation following membrane hyperpolarization and elevated intracellular Ca^2+^. In vivo studies that employed in vivo treatment with the K_Ca_3.1 channel blocker TRAM-34 and K_Ca_3.1 gene deletion or K_Ca_3.1 gene knockdown in cultured fibroblasts effectively reversed upregulated expression of fibrotic genes and reduced organ fibrosis [51,310]. Using mouse models of cardiomyopathy with severe fibrosis due to enhanced βAR stimulation by pharmacological or transgenic means, She et al. showed upregulation of Gal3 in inflammatory cells, fibroblasts and cardiomyocytes and that Gal3 promotes expression of K_Ca_3.1 as well as other inflammatory (TNF-α, MCP1, CD45, CD68, IL-6) and fibrotic genes (TGF-β1, CTGF, collagen) [51]. Recent studies have revealed that metabolic remodeling in heart disease is associated with upregulation and activation of both Gal3 and K_Ca_3.1 by metabolites such as α-ketoglutarate [310].

### 6.3. Interactions of Extracellular Gal3 and Galectin-3-Binding Protein

Gal3BP, also termed Lgals3BP or Mac-2-binding glycoprotein, is encoded by the LGAL3SBP gene [311]. Gal3BP is a highly glycosylated and secreted protein, detectable in most tissues and body fluids, and may be essential for biological actions of Gal3 [209,312]. The full-length Gal3BP protein consists of 585 amino acids, but its molecular weight is 90 to 100 kDa following extensive glycosylation at multiple potential N- or O-glycosylation sites. The Gal3BP structure consists of three functional domains: BACK domain, BTB/POZ domain, and SRCR domain [311]. Gal3BP is released through the canonical secretory pathway and co-release of Gal3BP with Gal3 was observed in extracellular vesicles [313]. Apart from Gal3 as its major receptor or ligand,, Gal3BP also interacts with galectin-1, β1-integrins and ECM proteins like collagen, fibronectin and laminin [314,315].

The majority of studies indicate disease-mediating roles of secreted Gal3BP in cancer [311,314,316] or inflammatory and fibrotic conditions [192,315,317,318,319,320]. Knockdown of either Gal3 or Gal3BP attenuates tumor growth and metastasis [311,321]. Thus, there is strong evidence for a synergistic role of Gal3–Gal3BP in tumorigenesis and metastasis [322,323,324], in which Gal3BP may enhance Gal3–EGFR signaling, leading to increased cMyc and epithelial–mesenchymal transition [321], and stimulate tubulogenesis, a cancer-promoting process that is VEGF-independent but Gal3-dependent [325]. Expression of Gal3BP is also induced by inflammatory molecules such as interferons, TNF-α or double-stranded RNA or DNA (poly I:C, dsRNA, dsDNA). Conversely, Gal3BP is able to interact with diverse extracellular and intracellular proteins, resulting in induction of interferons and pro-inflammatory cytokines. Such interaction has also been shown to enhance cell–cell adhesion and activation of inflammatory–signaling cascades. Gal3BP depletion attenuates hepatic fibrosis by reducing TGF-β1 availability and inhibiting genesis of hepatocarcinoma [318]. Through interacting with Gal3, circulating Gal3BP promotes the development atherosclerosis and induces plaque instability [312]. Gal3BP–Gal3 interaction has been shown to promote macrophage M1 polarization, causing endothelial dysfunction and pulmonary artery hypertension in an animal model of systemic lupus erythematosus [326]. Gal3BP is found to induce insulin resistance either directly by inhibiting insulin receptor phosphorylation and activity or indirectly via promoting the inflammatory TNF-α/TLR/NF-κB signaling pathway [327].

Both Gal3 and Gal3BP are simultaneously upregulated in settings of cancer or inflammation and co-exist extracellularly in exosomes or vesicles of human blood or body fluids [314,321,328,329,330] and are involved in inflammatory signaling and activation of fibroblasts through direct interaction [331,332]. In cancer patients, circulating Gal3BP presents either in the form of free protein or association with extracellular vesicles [311,333]. Elevated circulating levels of Gal3BP are also found in patients with carotid or coronary artery atherosclerosis [334,335], myocardial infarction [336], pulmonary hypertension [337], vascular diseases (e.g., systemic lupus erythematosus) [338,339], metabolic syndrome [320], or hepatic fibrosis of different etiologies [318,340,341,342]. Gal3BP levels predict severity of coronary atherosclerosis, risk of myocardial infarction and cardiovascular mortality [334,335,336,343], and such predictive power of Gal3BP is enhanced by the inclusion of Gal3 [336]. 

Given considerable similarities in biological actions between Gal3BP and Gal3, future study needs to explore the Gal3 dependency of currently known actions of Gal3BP in CVD. The mode of Gal3–Gal3BP molecular interaction also requires definition.

## 7. Summary and Future Research

### 7.1. Summary

In the last two decades, research on Gal3 has progressed significantly in the areas of transcriptional regulation, localization, protein modifications and intracellular actions, together with translational findings on Gal3 as a clinical biomarker and a disease mediator. Regulators or signaling pathways have been identified that play key roles in the regulation of Gal3 from transcription to localization-dependent biological actions. As discussed in this review, the functionality of Gal3 varies according to its intracellular or extracellular localization, quaternary structures with Gal3 oligomers or formation of Gal3 lattices with enhanced biological actions. Importantly, related to its localization, pleiotropic actions of Gal3 could be dependent on its CRD-binding capacity but also achieved by protein–protein interactions, particularly in controlling expression of genes, thereby regulating multiple signaling pathways critical for pathological events including cell proliferation, programmed death, inflammation, fibrosis and energy metabolism (Figure 7). Given the recent improvement in our understanding on the role of Gal3 in the development of CVD, Gal3 forms a critical therapeutic target. Whereas current development of Gal3 inhibitors focuses on CRD blockade (Table 1), other stages of Gal3 biosynthesis, modification and distribution also hold promise for effective interventions.

### 7.2. Broadening Anti-Gal3 Therapeutic Potentials

Limitations of CRD-targeted inhibitors. There is evidence that the function of extracellular Gal3 depends almost entirely on its CRD–glycoconjugate interaction, and thus carbohydrate ligand mimics would be suitable and effective inhibitors of Gal3 (Table 1). Whereas intracellular function of Gal3 could be independent of its interaction with glycoconjugates, the CRD may still be involved in intracellular protein–protein interactions without involving glycoconjugate binding. Given the recognition of the significant intracellular function of Gal3 (Figure 7), the development of novel classes of Gal3 inhibitors is desirable. Ideal criteria of Gal3 inhibitors would be exhibiting high affinity relative to endogenous glycoconjugates, selective to Gal3 relative to other galectins, biochemically stable in vivo, and distributing not only in the extracellular space or cell surface membrane but also entering into intracellular or nuclear compartments and inhibiting protein–protein interactions. In fact, among current inhibitors, very few are non-CRD-targeted (e.g., MG-257, Table 1). Regarding current inhibitors, their capability of intracellular entry by passing cellular membrane remains unclear, the process requiring low-polar surface area, as shown by Stegmary et al. [48]. Indeed, Liu et al. tested BBB permeability of two inhibitors, FJMU1887 and TD139, which is a well-studied Gal3 inhibitor (Table 1), and found satisfactory permeability for FJMU1887 but very poor permeability for TD139 [302]. A potential solution for improving drug delivery intracellularly is the use of drug carriers or polymeric assembled micelles, the subject of future investigation. Relevant to this is the evaluation of the efficacy of existing and newly developed Gal3 inhibitors with special assays of cellular uptake and their intracellular bioactivities. Therapy with a combination of inhibitors against both C- and N-domains of Gal3 would be expected to achieve better efficacy.

Extracellular secretion and circulating level of Gal3. Activation of monocytes/macrophages by circulating Gal3 promotes expression and secretion of Gal3, forming a vicious cycle. Accordingly, under conditions of significant inflammation like myocardial infarction, myocarditis, sepsis or atherosclerosis, a circulating pool of Gal3 may reflect Gal3 secretion into the circulation. We showed that, in mice subjected to ischemia–reperfusion, there is increased expression of Gal3 as well as other inflammatory markers in circulating immune cells [104]. In human patients with atherothrombosis, plasma levels of Gal3 are positively correlated with markers that reflect activation of circulating monocytes, such as oxidative stress [27]. Studies have also revealed that enhanced sympatho-βAR signaling augments cardiac expression and secretion of Gal3, evidenced by the transcardiac Gal3 gradient, together with enhanced Gal3 expression by circulating inflammatory cells, both contributing to the overall Gal3 plasma pool [31,104]. In cultured breast cancer cells, a mechano-sensing mechanism triggers rapid secretion of Gal3 into the medium [196], albeit it remains undetermined whether expression of Gal3 is simultaneously enhanced by mechano-sensing stimuli. Furthermore, released Gal3 can be rapidly taken up by cancer cells, a process associated with facilitation of cell spreading and adhesion [196]. Given that mechanotransduction is a fundamental signaling mechanism for cell activation, migration, proliferation and metastasis, as well as for activation of fibroblasts and myocardial hypertrophy [344], further research is warranted to address the role Gal3 in the relevant biological and pathological events. This is even more likely considering that Gal3 is an integrin-associated protein that acts as a mechano-sensing molecule (Figure 6B). Collectively, several interventions that attenuate inflammation, mechano-sensing stimuli or βAR activity are expected to suppress extracellular release of Gal3.

**Figure 10:**
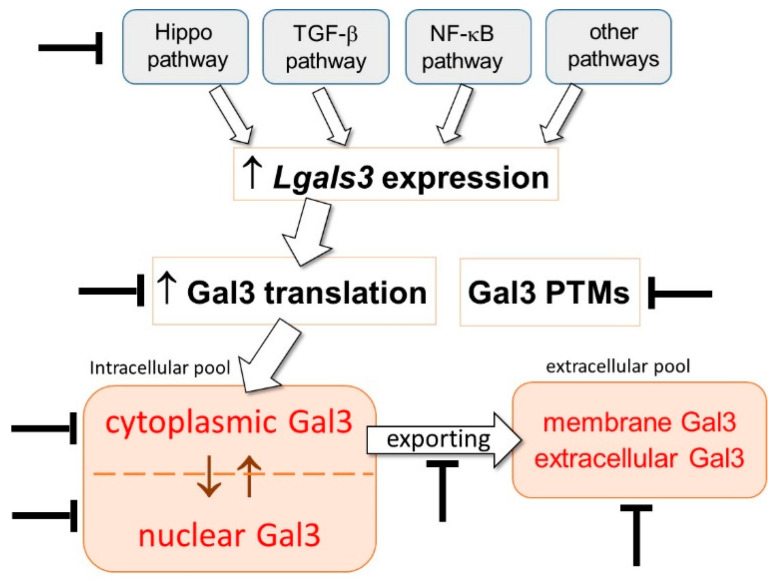
Targeted potential therapies that are independent of CRD blockade in the setting of cardiovascular disease. Along with CRD-targeted inhibitors to block Gal3 interactions with glycoligands, future research may focus on inhibition of *Lgals3* transcription, protein expression, post-translational modifications (PTMs) and intracellular or extracellular transport. Importantly, suppression of intracellular Gal3 actions would be expected to reverse dysregulated gene profiles, dysfunction of organelles, Gal3 intracellular signaling and extracellular export. ━┫ denotes inhibitory intervention.

Suppression of Gal3 expression or its intracellular activity. As reviewed in Section 2, other CRD-independent interventions would be expected to effectively inhibit Gal3 expression and activity under diseased conditions. Specifically, we highlight the transcriptional regulation of Gal3 expression as a promising therapeutic target to repress Gal3 upregulation commonly seen under pathological conditions (Figure 10). We speculate that suppression of Gal3 expression, with reduced intracellular and extracellular Gal3 levels, holds the potential to be developed for therapeutic use. Regulation of post-transcriptional modification of *Lgals3* (e.g., m6A) or PTM of Gal3 (e.g., phosphorylation) could form druggable strategies. Likewise, identification of nuclear localization and actions of Gal3 also suggests a further avenue for tuning the transcriptional regulation by Gal3, which contributes to the onset and progression of diseases.

### 7.3. Future Research Directions

First, Gal3 has long been the research focus in cancer and well identified as an oncogenic molecule (Figure 1). Higher levels of Gal3 expression were identified in numerous cancer types and associated with aggressive behavior and metastasis. By referring to studies on cancer tissues and cells, this review includes a significant body of advanced research findings on Gal3 biology, which is expected to inspire new research on Gal3–CVD. Notably, significant diversity exists among different cell types in Gal3 biology, such as expression level, intracellular distribution, shuttling and secretion. CVD involves distinct cell types contributing at different stages of pathologies. Hence, research is required to delineate the features of Gal3 in key cell types of cardiovascular tissues for better understanding of the role of Gal3 in promoting CVD. One example is the current appreciation of a pivotal role of macrophage-derived Gal3 under a variety of diseased settings. Aortic dissection is a critical clinical condition where cell types like macrophages, endothelial cells and VSMCs are sequentially activated, achieving persistent expression of Gal3 during the course of the disease. Lin et al. generated nanoparticles with triple elements of a Gal3-binding polysaccharide, a nitric-oxide-generating peptide and a hydrophobic drug carrier. When tested in vitro and in vivo, this nanomedicine enhanced uptake by lesion-contributing cells with regional accumulation and lowered the degree of aortic dissection, vascular inflammatory and degenerative phenotypes and reduced mortality [345].

Second, there is accumulating evidence for the dependency of Gal3 biological actions on its intracellular or extracellular localization (Figure 7) [108,193,194]. This issue is critical to our understanding of biological actions of Gal3 such as activation of fibroblasts, VSMCs or macrophages. Shirakawa et al. found that intracellular Gal3 effectively activates M2 macrophages whilst exogenously added Gal3 had no such activity [108]. This finding might explain in part the controversy on whether intracellular and extracellular Gal3 act similarly in activating macrophages or fibroblasts. Likewise, extracellular Gal3 promotes but intracellular Gal3 inhibits apoptotic cell death. Future research is needed to delineate biological actions of Gal3 in relation to its localization. Diverse findings exist on the efficacy of MCPs in a range of CVDs, including a clinical trial on hypertensive patients [49], showing either a significant inhibitory effect (e.g., against inflammation, fibrosis, hypertrophy and cardiac dysfunction) or lack of efficacy [32,49]. In the Mst1-cTG DCM mouse model, the ineffectiveness of treatment with MCPs was contrary to the significant benefits by a partial or complete deletion of the *Lgals3* gene [32], indicating a pivotal role of intracellular Gal3 in disease progression. Relevant to this is our limited understanding of the dissociation between the expression and secretion of Gal3 [49,104]. It has been shown that the anti-cancer efficacy of MCPs is more potent in small versus large products, likely due to different capacities for intracellular entry [346]. Another relevant research area is the regulatory mechanism by which Gal3 shuttles between the cytoplasm and nucleus or secretes extracellularly, processes likely forming therapeutic targets (Figure 10).

Third, the onset and progression of CVD involve phenotypic alterations and accompanied metabolic switching of diverse cardiovascular cell types. Future investigation is needed on the regulation by Gal3 of metabolism and the mechanisms involved. Intracellular Gal3, particularly mitochondria and the nucleus in localization, is of special significance given their direct and indirect (i.e., transcriptional regulation) influence on cellular metabolism (Figure 9). Actions of mitochondrial Gal3 affect not only cellular apoptosis and autophagy but also other functions, including energy metabolism, ROS generation, intracellular Ca^2+^ handling and coordination among organelles that participate in cellular metabolism (Figure 7). Influence by Gal3 on interactions among diverse organelles, particularly mitochondria and the endoplasmic reticulum, needs to be explored. In addition, the possible contribution of *Galig*-encoded mitogaligin in CVD remains to be tested.

Fourth, the formation of the Gal3–glycoconjugate lattices is dependent not only on the expression and secretion of Gal3 but also on the density of glycosylated ligands at the membrane that form branches with Gal3 oligomers (Figure 2C). Heart disease is associated with metabolic remodeling manifested by enhanced aerobic glycolysis and glutaminolysis, which would be expected to inhibit the glucose or glutamine-driven biosynthesis of N-acetylglucosamine (GlcNAc), thereby weakening the strength of Gal3–glycoconjugate lattices. In other words, aerobic glycolysis and glutaminolysis serve as critical negative regulators of Gal3 lattice strength. Further research is warranted to explore the turnover of glycoconjugates and the role GlcNAc salvage in relation to Gal3 lattice formation. There is emerging evidence that metabolic alterations regulate membrane glycan ligands and hence the formation of Gal3 lattices [151]. Another key component of cardiac metabolic remodeling involves abnormalities in membrane lipid composition, including glycophospholipids, glycosphingolipids and glycoceramides [283], which would be expected to alter lattice formation via Gal3–glycolipid interactions. It is also likely that Gal3–lipid interactions at the cellular membrane may alter the turnover rate of certain classes of structural lipids [131,184,284]. It is noteworthy to point out that the direct viewing of such highly dynamic Gal3 lattice structures remains to be achieved.

Finally, augmented expression of Gal3 occurs in settings of cancer and CVD, where Gal3 acts as a key player as a biomarker and disease mediator. Hence, Gal3 might contribute significantly to mutual comorbidity or cardio-oncology, which deserve further investigation. There is also evidence that the expression and function of Gal3 are influenced by commonly used medications for either CVD or cancer [347,348,349]. Research may focus on well-studied molecules known to be critical in both diseased settings. One example is YAP which is well known as a cancer promoter whilst its activity in cardiomyocytes is beneficial. Many anti-cancer drugs would suppress the transcriptional activity of YAP, leading to anti-cancer efficacy, whilst this effect, when occurring in the myocardium, would result in cardiotoxicity and cardiomyopathy due in large part to mitochondrial damage and cell death [96,350]. Another case is the potential interplay between p53 and Gal3 in CVD. p53 is a well-known tumor suppressor that induces cell apoptosis and senescence and also regulates metabolism [351]. In cancer tissues, there exists reciprocal inhibition of p53 and Gal3 by which p53 suppresses cancer growth in part through antagonizing the pro-oncogenic action of Gal3. Interestingly, p53 has recently been shown to contribute to organ fibrosis [352], but the influence by Gal3 in this setting is not studied.

## Figures and Tables

**Table 1:** List of current Gal3 inhibitors with their Kd value and nature of chemicals.

Name	K*d* or IC_50_	Nature of Chemical
CB1107	IC_50_ = 37 nM (hGal3)	Small molecule glucopyranoside
C3-C12	K*d* = 70–88 nM	Synthetic peptide targeting CRD
Compound 5 (Gal3-IN-4) Compound 9 (Gal3-IN-2) Compound 29 (Gal3-EGFR-IN-1) Compound 53 (Gal3-IN-1)	IC_50_ = 21 nM (hGal3), 167 nM (mGal3) IC_50_ = 8.3 μM K*d* = 52.3 μM K*d* = 4.1 μM	Synthetic carboxamide analogs, CRD ligands
Davanat	K*d* = 68 nM	Polysaccharide-derived CRD ligand
FJMU1887	K*d* = 1.55 μM	Synthetic small molecule against CRD
GB0139 GB1100 GB1107 (selvigatin) GB1211 (modified galactose) GB1497	K*d* = 14 nM K*d* = 37 nM K*d* = 37 nM K*d* = 25 nM K*d* = 2.7 μM	Synthetic monosaccharide, CRD ligands
GCS-100	not specified	Natural plant-derived polysaccharides, CRD ligand
GM-CT-01 (davanat)	K*d* = 200 μM	Natural plant-derived CRD ligand
GR-MD-02 (belapectin)	K*d* = 2.9 μM	Natural plant-derived CRD ligand
HH1	K*d* = 38 μM	Safflower-derived arabinogalactan, CRD ligand
Lactose	K*d* = 1000 μM	Natural plant-derived CRD ligand
LacNAx	K*d* = 200 μM	Natural plant-derived CRD ligand
MG-257	K*d* = 0.15 μM	Small molecule, non-CRD targeted
Modified thiodigalactoside	K*d* = 1.6 nM	Synthetic disaccharide, CRD ligand
Modified citrus pectin	not specified	Natural polysaccharides, CRD ligand
Modified LacNAc	K*d* = 0.88 μM	Synthetic disaccharide, CRD ligand
N-Acetyllactosamine (LacNAc)	K*d* = 67 μM	Natural plant-derived oligosaccharides, CRD ligand
Pectasol	K*d* = 37 nM	Natural plant-derived CRD ligand
PTX-013	K*d* = 5.3 μM	Small molecule targeting CRD binding
TB006	Not specified	Monoclonal antibody against CRD
TD39 TD139	K*d* = 36 nM K*d* = 68 nM	Small molecule obtained by MedChemExpress and AOBIOUS
Thiodigalactoside (TDG)	K*d* = 49 μM	Natural plant-derived disaccharide, CRD ligand
TDG-BSA conjugate 11 TDG-BSA conjugate 12	IC_50_ = 19 nM IC_50_ = 1.9 nM	Thiodigalactoside conjugated to bovine serum albumin, CRD ligand

Chemicals are listed in alphabetic order and also grouped based on developers. Abbreviations: K*d*: dissociation constant, IC_50_: effective concentration for 50% response. mGal3 and hGal3: mouse or human Gal3.

## Data Availability

Data available in a publicly accessible repository.

## Abbreviations

AGE: advanced glycation end-product; AP-1, activator protein-1; βAR, β-adrenergic receptor; Bcl-2, B cell lymphoma-2; CK1, casein kinase 1; CRD, carbohydrate recognition/binding domain; CREB, cAMP-responsive element-binding protein; CTGF, connective tissue growth factor; CVD, cardiovascular disease; DAMP, danger-associated molecular pattern; DCM, dilated cardiomyopathy; ECM, extracellular matrix; EGFR, epithelial growth factor receptor; EMT, epithelial–mesenchymal transition; Gal3, galectin-3; Gal3BP, galectin-3-binding protein; GALIG, *Lgals3* internal gene; Glut, glucose transporter; HIF-1α, hypoxia-inducible factor-1α; ICAM, intercellular adhesion molecule; IL, interleukin; INF-γ, interferin-γ; KO, knockout; K_Ca_3.1, intermediate-conductance Ca^2+^-activated K^+^ channel; LLPS, liquid–liquid phase separation; LPS, lipopolysaccharide; MAM, mitochondria-associated membrane; MCP, modified citrus pectin; m6A, N6-methylaadenosine; MCP1, monocyte chemoattractant protein 1; MMP, matrix metalloproteinase; MRTFA, myocardin-related transcription factor A; Mst1, mammalian sterile 20-like kinase1; NF-κB, nuclear factor kappa-light-chain-enhancer of activated B cells; NLR, Nod-like receptor; NLRP3, NLR family pyrin domain containing 3; NOX, nicotinamide adenine dinucleotide phosphate oxidase; NPC, nuclear pore complex; PKM, pyruvate kinase M; PPAR, peroxisome proliferator-activated receptor; PTM, post-translational modification; ROS, reactive oxygen species; Runx, Runt-related transcription factor; TEAD, TEA-domain family member; TG, transgenic; TGF-β, transforming growth factor-β; TLR, Toll-like receptor; TRIM, tripartite motif family protein; UPR, unfolded protein response; VCAM, vascular cell adhesion molecule, VEGF, vascular endothelial growth factor; VSMC, vascular smooth muscle cell; YAP, yes-associated protein.
